# Analysis of the Growth of Hydrogel Applications in Agriculture: A Review

**DOI:** 10.3390/gels11090731

**Published:** 2025-09-11

**Authors:** Carolina Buitrago-Arias, Piedad Gañán-Rojo, Mabel Torres-Taborda, Luisa Perdomo-Villar, Catalina Álvarez-López, Natalia Jaramillo-Quiceno, Gustavo Adolfo Hincapié-Llanos

**Affiliations:** 1 Facultad de Ingeniería Agroindustrial, Universidad Pontificia Bolivariana, Circular 1 70-01, Medellín 050031, Colombia; carolina.buitrago@upb.edu.co (C.B.-A.); catalina.alvarezl@upb.edu.co (C.Á.-L.); natalia.jaramilloq@upb.edu.co (N.J.-Q.); 2 Facultad de Ingeniería Química, Universidad Pontificia Bolivariana, Circular 1 70-01, Medellín 050031, Colombia; piedad.ganan@upb.edu.co (P.G.-R.); mabel.torres@upb.edu.co (M.T.-T.); luisa.perdomo@upb.edu.co (L.P.-V.)

**Keywords:** hydrogel, integrated crop solutions, biostimulants, agriculture, fertilizer, herbicide, soil, PRISMA, bibliometric analysis, systematic review

## Abstract

Feeding a growing population under the pressures of climate change requires solutions that safeguard yields while strengthening agricultural resilience. Integrated Crop Management (ICM)—which combines precise fertilization, efficient water use, and targeted pest control—offers a promising framework. Hydrogels, with their water retention and controlled release properties, can enhance ICM by improving fertilizer efficiency, reducing water loss, and supporting soil health. Despite extensive research, their optimal use in agriculture remains unclear, and limitations continue to restrict large-scale adoption. To address this gap, this study applies the Preferred Reporting Items for Systematic Reviews and Meta-Analyses (PRISMA) methodology alongside bibliometric analysis to examine hydrogel applications in ICM from 2000 to 2024. Ninety Scopus-indexed publications were analyzed across four domains: pesticides, nutritional growth inputs, soil conditioners, and bioactive substances. The results reveal a marked increase in hydrogel structural complexity, greater diversity in characterization methods, ongoing reliance on high-impact pesticides despite advances in bio-based hydrogels, and persistent gaps in assessing environmental impacts and regulatory compliance. These findings underscore the need for stronger collaboration between academia and industry to translate hydrogel research into effective, sustainable agricultural practices under changing climatic conditions.

## 1. Introduction

Plant diseases, soil salinity, drought, and extreme temperatures are increasing challenges to agricultural systems [1,2,3]. Biotic and abiotic stress considerably diminish agricultural output, with disease-related losses estimated to range from 20% to 40% [4]. In addition to diminishing productivity, they impair soil health, undermine crop quality, and jeopardize global food security [5]. Confronting these challenges needs novel agricultural practices that improve crop resilience and maximize resource efficiency.

Integrated Crop Management (ICM) is a holistic strategy that enhances agricultural productivity through the integration of precision fertilization, water management, and targeted pest control. The Food and Agriculture Organization (FAO) identifies ICM as a crucial strategy for sustainable agriculture, promoting soil health, optimizing water-use efficiency, and increasing crop yields by 10–14% compared to traditional practices [6]. Furthermore, ICM advocates for conservation agriculture by enhancing soil aggregation, improving water infiltration, and optimizing the controlled release of nutrients and agrochemicals [6].

ICM promotes a balanced and sustainable agroecosystem by integrating inputs such as fertilizers, plant protection agents (e.g., insecticides), and biostimulants derived from organic compounds, microorganisms including fungi [7], algae [7,8], inorganic compounds [7,9], and biopolymers like chitosan [10,11]. The significance of utilizing biostimulants pertains to their contribution to enhancing tolerance to abiotic stress, improving quality features, or facilitating the availability of limited nutrients in soil or the rhizosphere [12].

Persistent challenges remain regarding efficacy, particularly volatilization, degradation, and inadequate control over the release of fertilizers, plant protection agents, or biostimulants, necessitating the integration of these compounds into with other components or delivery systems [13,14,15]. In this context, polymer hydrogels emerge as a promising strategy, offering not only controlled release but also the capacity to alleviate soil-related problems such as soil compaction, erosion, and water runoff [3,16].

Hydrogels are versatile three-dimensional (3D) network structures formed by a monomer or polymer through a gelation process [17]. Generally, it can absorb substantial quantities of water without disintegrating or dissolving [18]. The 3D network can be created through physical crosslinking facilitated by non-covalent bonds, including hydrogen bonding, van der Waals forces, or physical interactions [18]; or through chemical crosslinking, where covalent bonds are essential [16,18]. Their agricultural relevance derives from their high water-holding capacity, which can alleviate drought stress and soil erosion while enabling the controlled release of agrochemicals, thereby enhancing plant water availability and nutrient uptake [19]. Hydrogel formulations have the potential to enhance soil aeration, microbial activity, and root development, which may lead to improved crop performance [16].

Hydrogels can be synthesized from various sources, including natural polymers such as chitosan and synthetic polymers like Polyvinyl Alcohol (PVA) [20]. Moreover, combinations of natural and synthetic polymers, such as PVA/sericin, are also feasible [21]. Additionally, polymer composites or nanocomposites, based on bacterial nanocellulose, can be recognized for these applications [22]. Resolving technical issues related to hydrogel structure, interactions with agricultural inputs, soil type, and environmental conditions is essential for the successful integration of hydrogel-based systems. This situation prompted extensive global research focused on the formulation and evaluation of hydrogels. Significant aspects of hydrogel structure have been extensively reviewed in the literature, including the sources of hydrogel materials, crosslinking processes that influence overall performance, and physical evaluations addressing absorption and desorption characteristics [18,23,24].

Nevertheless, these assessments have primarily concentrated on structural and physicochemical attributes, without providing enough connection to comprehensive agronomic frameworks such as Integrated Crop Management (ICM). The absence of integration hinders the optimization of hydrogels for field conditions and the exploitation of potential synergies with fertilizers, insecticides, and other agricultural inputs. Moreover, insufficient emphasis has been placed on identifying the variables that limit the widespread adoption of hydrogels, along with their ramifications for sustainability and the circular economy. Investigating the progression of this thematic domain enables the identification of trends and the specification of priority elements that should guide future research aimed at enhancing these applications. This work addresses this gap by integrating a systematic literature review, conducted in accordance with the Preferred Reporting Items for Systematic Reviews and Meta-Analyses (PRISMA) criteria, with bibliometric analysis. This dual methodology facilitates the identification of research trends, thematic clusters, and underexplored areas, establishing a systematic foundation for advancing hydrogel applications in agriculture. Although both PRISMA and bibliometric techniques are widely applied in materials research [25,26], their combined use in the context of hydrogel-based agricultural solutions has not been previously reported, representing a novel contribution. To operationalize this approach, this study examined 90 papers published between 2000 and 2024, indexed in the Scopus database, across four primary domains: pesticides, nutritional growth inputs, soil conditioners, and bioactive compounds. The search strategy targeted studies on chemical substances or agricultural inputs that enhance plant health or improve soil properties. The methodology used to select publications that met the search equation and the inclusion/exclusion criteria is detailed in Section 4. The results provide valuable insights for both early-stage and advanced investigations aiming to combine hydrogels with complementary materials for ICM or other sustainable agricultural practices. This research is relevant to large-scale production systems as well as small-scale contexts, including urban agriculture, where resource optimization and climate resilience are critical. Furthermore, it may support both emerging and established investigations seeking to explore the advantages of hydrogels combined with substances, inputs, or payloads—such as nutrients, agrochemicals, active agents, or biostimulants—applicable to strategies that promote ICM or similar approaches aimed at advancing sustainable agricultural practices.

## 2. Results and Discussion

### 2.1. Macro- and Micro-Level

**Figure 1:**
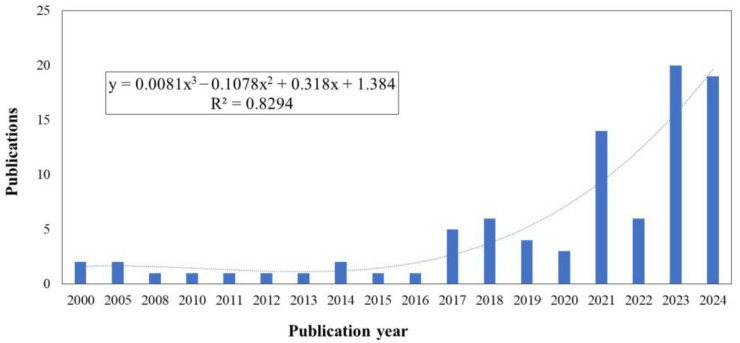
Annual number of publications on hydrogel applications in agriculture (2000–2024).

Grounded in the study’s scope—encompassing both large-scale production systems and small-scale contexts such as urban agriculture, and emphasizing hydrogels in association with nutrients, agrochemicals, active agents, and biostimulants within Integrated Crop Management (ICM) strategies—the results are structured to first provide a macro-level assessment of research evolution, followed by a micro-level analysis of individual contributions. This structure, enabled by the combined application of the Preferred Reporting Items for Systematic Reviews and Meta-Analyses (PRISMA) methodology and bibliometric analysis, provides a comprehensive framework for identifying publication trends, thematic clusters, and research gaps. The macro-level overview begins with an examination of publication growth patterns over time, offering context for subsequent in-depth analysis at the document level. As shown in Figure 1, the documents analyzed in this work cover the years 2000 to 2024. The earliest publications include to the works of the Saraydın et al. (2000) [27] which focused on the use of the hydrogel in pest control by evaluating the controlled release of the water-soluble agrochemical herbicide such as sodium 2,2 dichloropropionate (Dowpon). In the same year, Karadağ et al. (2000) [28] explored applications in crop nutrition by the analysis of the interaction between the hydrogel and fertilizers such as ammonium nitrate, potassium nitrate and ammonium sulfate. They also evaluated the potential for the pest control of the grasses and weeds using Dalapon. Both studies employed the same type of the pest control approach, involve the **same** active **compound**, and utilized hydrogels are based on acrylamide.

Figure 1 illustrates the steady increase in publications on this topic, with 2023 representing the most active year. The growth trend was modeled using a cubic polynomial regression (y = 0.0081x^3^ − 0.1078x^2^ + 0.318x + 1.384), which yielded an R^2^ value of 0.8294. In the physical sciences and engineering disciplines, R^2^ values above 0.70 are generally regarded as acceptable indicators of fit, providing meaningful descriptive insight into data trends [29]. Although this model is not intended as a predictive tool, it effectively summarizes the observed growth—particularly between 2020 and 2023—and suggests that publication activity in this field is likely to continue increasing over the next five years. This macro-level overview sets the stage for the detailed bibliometric analysis that follows, which integrates both macro- and micro-level perspectives to identify key institutions, leading authors, and thematic keyword patterns shaping research in this domain.

**Figure 2:**
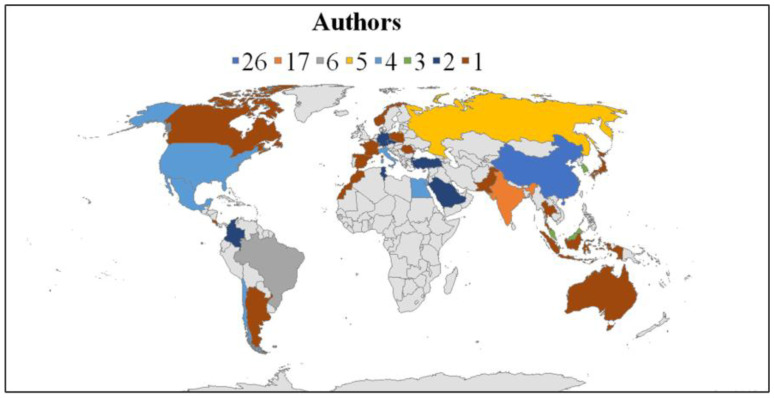
Geographical distribution of authors based on Scopus-indexed publications (2000–2024).

Following the upward publication trend shown in Figure 1, the macro-level analysis also examined the geographical distribution of research activity. As shown in Figure 2, contributions originate from 31 states across multiple regions, reflecting the widespread scientific interest in hydrogel applications for agriculture. This global distribution provides a basis for examining the relative research intensity of individual countries, offering insight into why certain regions demonstrate higher activity levels than others. The highest proportion of documents (29%) were authored by researchers based in China, followed by those in India (19%), and Brazil (7%). This point is noteworthy, given that these three emerging market economies have significantly expanded their agricultural activities over the past two decades and play a major role in agricultural trade policy issues [30,31]. In the case of China, the earliest publication identified corresponds to the work of Liu et al. (2005) [31], which explored the use of sodium carboxymethylcellulose hydrogel to improve water absorption However, until the continuous publications are observed since 2020, and the most productive year corresponds to 2024 with seven works. The trends observed in these documents correspond to more interest in the last year on delivery efficiency of compounds, especially on fertilizers [32,33]. Also, it is observed an interest on the water retention considering aspects such as soil moisture management [32,34], and in some cases are linked to the use of the humidic substance, especially considering soil fertility by use of the fertilizers [35]. and water retention [36].

In the case of India, the first documented identified in this work corresponds to Kumar and Kaith (2010) [37] that are focused on the develop of the hydrogel based on Psyllium/acrylic acid for the release of fungicide as copper sulfate that is useful in the control of the multiple fungi and bacteria that promote plant diseases. As similar in the case of the documents published by Chinese authors is observed interest by the compound control release, and the use of natural polymers or raw materials to develop the hydrogel. In the case of Brazil, the first document identified corresponds to Tanaka et al. (2021) [38] work where is observed again a concern related to the compound release, as the commercial product herbicide dibromide monohydrate considering a bio-based nanocomposite. In this work the trends observed are linked to the developed hydrogel based on natural resources supporting also the developed ecofriendly materials, as well as a more interest in advanced functionalization of the hydrogels. Tendency that is observed also in the case of the Chinese and Indian works.

Additionally, it is important to note that certain states participating in these challenges are not solely middle-income but also serve as importers and exporters of agrochemicals, such the Russian Federation [39], Mexico [40], and Argentina [41]. This geographical profile of research activity provides the foundation for narrowing the focus to the micro-level, where the analysis examines the institutions and authors driving research in this domain.

In total, 160 authors were affiliated with 158 organizations, with 60% based in university departments, and 2% in industry; the remaining institutions comprised research and development institutes or centers supported by universities or national governmental entities. Notably, industry-affiliated authors consistently collaborated with academic institutions [2,42]. This represents an opportunity to further promote interinstitutional collaboration, a factor of particular relevance for advancing the integration of hydrogels to the ICM practices.

**Figure 3:**
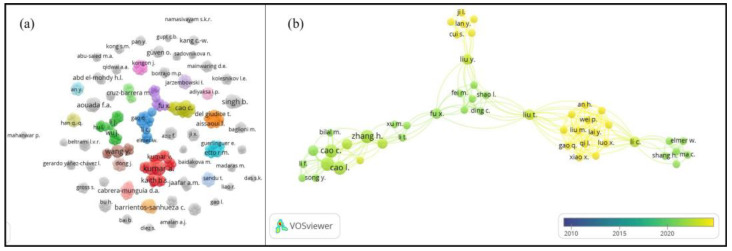
Authorship analysis: (**a**) distribution of authors; (**b**) collaboration networks among authors. Elaborated using VOSviewer.

Building on the importance of inter-institutional collaboration for advancing the integration of hydrogels into ICM practices, the author network analysis provides further insight into the dynamics of research partnerships. As shown in Figure 3a, the non-gray colored clusters represent groups of authors collaborating across different documents, while the gray clusters indicate isolated author groups working independently. Overall, the observed level of collaboration is relatively low. This situation can be linked to the diversity of the focus on the topics, motivated also by the type of hydrogels produced and the necessities to evaluate their efficiency on ICM strategies. Strengthening cross-institutional and interdisciplinary networks, particularly those connecting research teams working on complementary aspects of hydrogel design, evaluation, and field implementation, could enhance knowledge exchange and accelerate the translation of laboratory findings into practical agricultural applications. This need for stronger collaboration is further reflected in the temporal pattern of existing partnerships. As shown in Figure 3b, collaborations among the most active clusters identified in Figure 3a have been recorded only since 2018, underscoring both the novelty of the topic and the opportunity to expand cooperative research efforts. In particular, several works involving authors from industry also included participation from academic institutions, highlighting the importance of cross-sector collaboration in advancing hydrogel research for agricultural applications. Complementing this collaboration analysis, citation data provide another perspective on the field’s maturity and influence. The Scopus database yielded a total of 1,816 citations, with 91% of papers cited at least once—reinforcing the previously noted relevance of this research domain. Notably, 63% of the non-cited documents were published in 2024, reflecting the short time available for citation accumulation. The most referenced document, “Guar gum-crosslinked-soya lecithin nano-hydrogel sheets as effective adsorbent for the removal of thiophanate methyl fungicide” [43], has received 118 citations. This study offered novel insights into the physical interaction mechanisms between thiophanate-methyl fungicide and the synthesized hydrogel, highlighting potential applications in water detoxification methods to address pesticide contamination.

**Figure 4:**
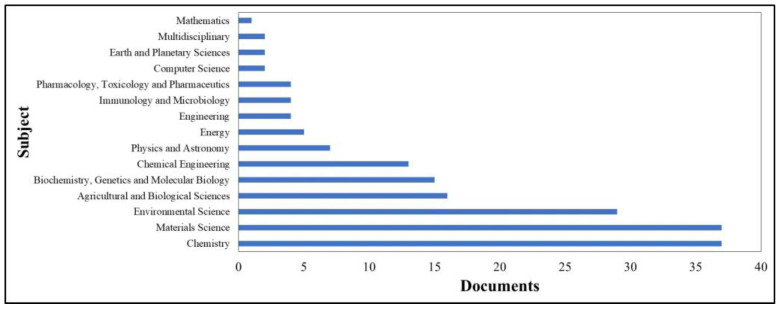
Distribution of documents across subject areas (2000–2024).

With regard to the journals preferred by the authors, a total of 63 journals were identified, spanning 15 subject areas, as shown in Figure 4. These results suggest a notable degree of interdisciplinarity within the topic. However, a clear preference is observed for topics related to materials and polymer science. For example, the journal with the highest number of documents is Carbohydrate Polymers with 6 documents, followed by International Journal of Biological Macromolecules and Journal of Polymers and the Environment, both with 4 documents.

**Figure 5:**
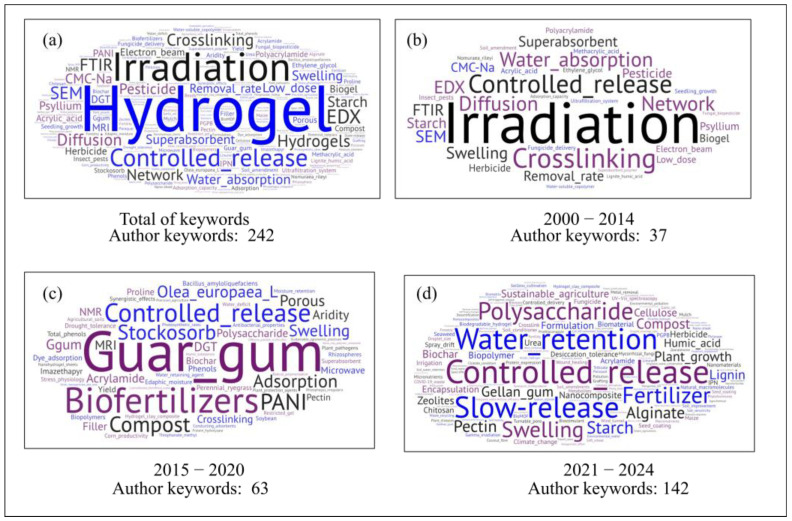
Temporal variation in author keywords illustrating the evolution of research themes, where (**a**) presents the overall frequency of keywords, (**b**) highlights terms used between 2000 and 2014, (**c**) depicts those from 2015 to 2020, and (**d**) reflects keywords from 2021–2024.

From the distribution of journals, the analysis then shifts to the thematic content of the publications, as reflected in the authors’ chosen keywords. A total of 242 author keywords were detected across the studied articles, as shown in Figure 5a. As expected, the most frequently mentioned author keyword is “hydrogel.” However, as in other cases, the studies also reference specific hydrogel types, including bio-based substances [44,45,46] or nanocomposites [38,46,47,48,49], as well as variations in crosslinking processes, ranging from beam-based methods such as gamma radiation [50] to more conventional chemical approaches using the crosslinker N,N′-methylenebisacrylamide (MBA) and ammonium persulfate (APS) as the initiator [51].

Figure 5b–d depict the temporal evolution of the contextual focus. To improve the interpretability of this analysis, the term hydrogel was excluded. Although the number of words increased, it was possible to observe the inclusion of more bio-based components during the period 2015–2020, such as Guar gum (Figure 5c), or the starch or other polysaccharides (Figure 5d). As shown Figure 5b–d, there is clear evidence of an evolution in applications. For instance, in the most recent works (Figure 5d) the keywords include terms such as “sustainable agriculture” [52]. In this same period the use of natural polymers is remarkable in order to produce more eco-friendly materials [53,54,55].

With regard to controlled release, an increase in publications has been observed, particularly those addressing fertilizer [34] or pesticides [56]. One of the most notable aspects concerns the advancement of the precision input delivery [57]. However, no specific use of the term associated with IMC was observed. This may be attributed to only 26 works (29%) in which there is a clear notice of collaboration between authors from departments in materials or chemical science and those in agricultural fields. This highlights an excellent opportunity to foster more interdisciplinary research.

**Figure 6:**
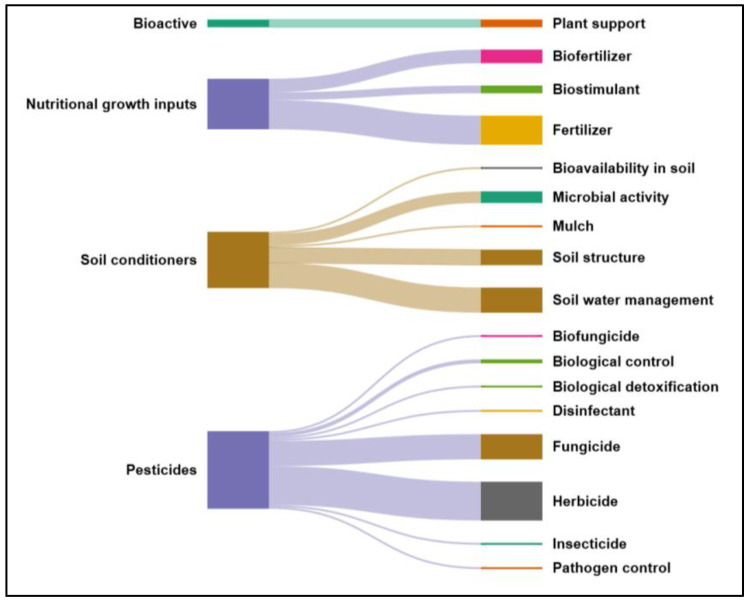
Sankey diagram of the distribution categories and subcategories identified. Image created using SankeyMATIC.

From Figure 5, author keywords highlight applications related to ICM practices, such as water retention for soil improvement and the use of biofertilizers and fertilizers, even when not explicitly stated. The preceding analyses provide the contextual foundation for a more detailed examination of hydrogel applications, which are classified into four categories: bioactive products, nutritional growth inputs, pesticides, and soil conditioners (Figure 6). The Sankey diagram illustrates their distribution, with most publications addressing pesticides, followed by soil conditioners, and fewer on bioactive products, which enhance plant tissue growth and support controlled bioactive release [58]. Six studies were classified into more than one category.

### 2.2. Analysis of Categories

A focused analysis of these four thematic groups provides critical insight into how hydrogels are being positioned within agricultural systems. By separating the publications into categories—bioactive products, nutritional growth inputs, pesticides, and soil conditioners—it becomes possible to trace specific research trends, identify methodological approaches, and highlight persistent challenges. This categorization not only clarifies the breadth of applications but also underscores the need for targeted innovation to align hydrogel development with practical agronomic demands. The discussion proceeds according to prevalence in the dataset, beginning with pesticides and concluding with bioactive products, which, although less represented, demonstrate noteworthy potential for plant growth promotion and controlled release of active compounds.

#### 2.2.1. Pesticides

Pesticides are important in agriculture as they effectively enhance crop yields by repelling, eradicating, or mitigating the impact of pests. Their continued usage can lead to significant issues, such as environmental pollution and health hazards [59], which hydrogel-based remedies may address. This section examines various methodologies identified in the selected texts.

As previously mentioned, this category contains the highest number of documents—38 in total. Table S1 provides details on the types of hydrogels developed, the pesticides loaded, the crosslinking processes employed, and the methods used for hydrogel characterization. As shown in Figure 6, the identified subcategories include biofungicide, biological control, biological detoxification, disinfectant, fungicide, herbicide, insecticide, and pathogen control. Despite this variety, there is a clear trend toward developing environmentally friendly formulations. This tendency is reflected in the use of biodegradable materials for hydrogel production, to support the active compounds. Examples include sodium alginate (SA) and carboxymethyl cellulose (CMC) [60], gellan gum [61], and carboxymethyl chitosan (C-CS)/sodium alginate [62]. Another example is an alginate/amidated pectin hydrogel, which has been used to encapsulate the biocontrol microorganisms, including *Trichoderma koningiopsis* Th003 [63].

**Figure 7:**
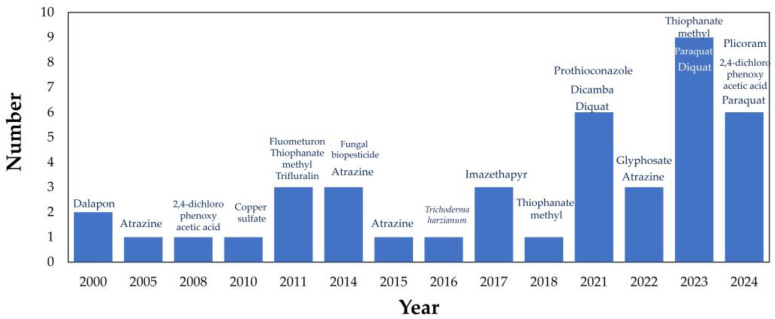
Temporal evolution of pesticide types reported in the analyzed documents.

Figure 7 illustrates the evolution of the pesticides examined by the authors. Despite this trend, highly toxic substances such as paraquat and copper sulfate continue to be referenced in certain documents—likely due to their increasing use, particularly paraquat in 2024, given its high efficacy in weed control [64]. Novel hydrogel-based systems have been reported, including the study conducted by Dong et al. (2023) [53], which produced a metal–organic framework (MOF) nanoparticle synthesized from FeCl_3_ and 2-aminoterephthalic acid to address challenges associated with its application. The hydrogel material contains mesoporous crystalline cages that functioned as carriers for herbicide encapsulation. Additional studies have investigated nanocomposites—comprising methylcellulose or chitosan reinforced with zeolite and further enhanced by poly(methacrylic acid)-co-polyacrylamide—to mitigate or diminish the leakage of hazardous compounds, even under fluctuating pH circumstances. [56].

Other commonly used chemicals, which remain toxic yet less hazardous than paraquat and copper sulfate, are also documented in Table S1 and Figure 7. These include Dalapon (sodium 2,2-dichloropropionate) [28], atrazine [51,65,66,67], and glyphosate [68]. In several cases, the primary objective was to achieve the controlled release of these compounds under specific conditions, particularly in aqueous environments [69].

Alongside strategies aimed at improving controlled release and reducing the adverse effects of pesticides, the adoption of eco-friendly or biologically based agents is also apparent. This includes fungal biopesticides [70], copper oxide nanoparticles that demonstrate effectiveness against various plant pathogens [71], garlic oil [46], and plant-derived compounds such as glycoalkaloids isolated from tomato and potato foliage [60].

**Figure 8:**
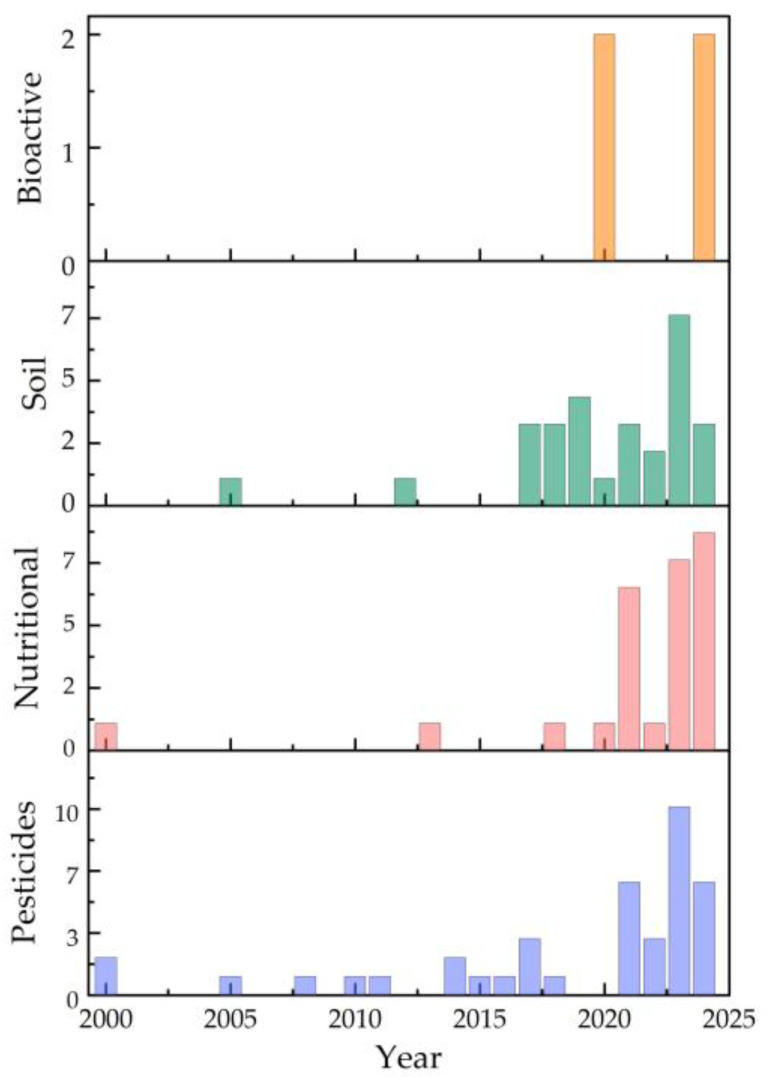
Temporal evolution of hydrogel material types reported in the analyzed documents.

The reviewed documents reveal a wide variety of hydrogel materials, as shown in Table S1, with 38 distinct types identified and a marked increase in diversity since 2017, as illustrated in Figure 8. Although many types were reported, a substantial proportion of formulations were based on acrylic acid, such as psyllium/acrylic acid [36], methacrylic acid [38,49,56,72], sodium alginate [60,62,73], and bio-based materials including chitosan [62,71,74] and starch [67,70,72]. The hydrogels identified include traditional copolymers such as polyacrylamide/poly(ethylene oxide), as well as semi-interpenetrating networks (semi-IPNs) based on synthetic polymers [65] and others incorporating natural polymers such as C-CS/SA [62]. Additionally, there is growing development of composites and nanocomposites that integrate inorganic nanostructures such as nanoclays [66], zeolites [37,49,56], copper oxide [71], and even nanocellulose crystals [75].

**Figure 9:**
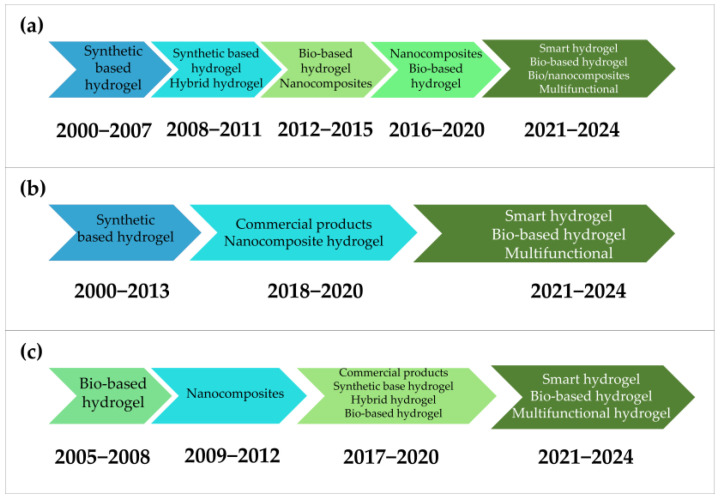
Evolution of hydrogel material types: (**a**) pesticides, (**b**) nutritional growth inputs, (**c**) soil conditioners.

A notable trend is the increasing investigation of hydrogels derived from composites and biocomposites. In summary, regarding the types of materials, as illustrated in Figure 8 and Figure 9a, this diversity can be categorized as synthetic polymers [76,77], bio-based polymers. Some of these materials are further classified as smart hydrogels due to their ability to respond to external stimuli such as variations in pH or controlled water retention and release [57]. However, in the most recent period examined in this study, certain hydrogels have been considered as multifunctional, owing to the incorporation of diverse components and their capacity to support and adapt to multiple environmental stimuli. These functions include water absorbency at different pH, temperature, and light levels [78], as well as the controlled release of compounds from microbeads, for example, those based on Ca-alginate graft copolymer of poly[N-isopropyl acrylamide-co-N,N-diethylacrylamide] [79].

**Figure 10:**
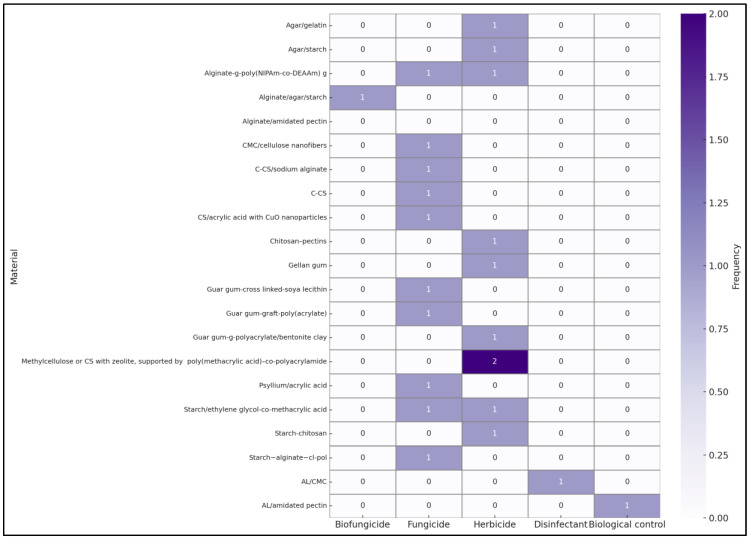
Document frequency linking bio-based hydrogels with pesticide categories. Values denote the number of studies reporting each case. Prepared with the assistance of artificial intelligence (ChatGPT, OpenAI, 2025), based on data and instructions provided by the authors.

Alternatively, as illustrated in Figure 10, which aggregates 21 studies featuring natural or biodegradable components, it becomes evident that there is no consistent correlation between the hydrogel type employed and the pesticides incorporated. The numbers in the figure represent the frequency of documents reporting a specific combination of hydrogel material and pesticide category. The absence of correlation arises because hydrogel selection is typically based on general properties—such as biodegradability, swelling capacity, or mechanical stability—rather than the nature of the active substance. For example, hydrogels based on chitosan (CS) have been used for both fungicides [11,62,71] and herbicides [49,56,74], while alginate (AL)-based hydrogels have been applied across all subcategories [60,68,70,73,80,81]. This finding confirms that multifunctional hydrogels are not strictly linked to a particular pesticide group.

Several authors have emphasized that the structural properties of hydrogels are essential for the regulated release of compounds [18,23,82]. Key factors include the polymer backbone, the integration of fillers—such as those utilized in composites and nanocomposites—and the density of network crosslinking [82]. The release profile can be significantly influenced by molecular interactions between the hydrogel matrix and the encapsulated substances [82]. Variations in crosslinking density during hydrogel synthesis may modify the release rate of compounds while concurrently influencing attributes such as water retention and soil-holding capacity [82]. This indicates that in developing novel hydrogel, it is essential to systematically evaluate the influence of these critical parameters on both compound absorption/release dynamics and water absorption/release characteristics.

Multiple techniques for crosslinking have been identified (see Table S1). The predominant technique involves chemical crosslinking using N,N′-methylenebisacrylamide (MBA) as a crosslinker and ammonium persulfate (APS) as an initiator. This strategy has been reported in several studies [51,69,71,83,84] and remained widely applied in works of 2024 [57]. The integration of MBA and APS is extensively utilized owing to its straightforwardness, repeatability, and tunable gel characteristics. This technique facilitates the formation of resilient hydrogel networks and offers operational simplicity, rendering it highly suitable for agricultural applications, especially in controlled pesticide release systems. In the context of APS, this initiator has been employed in conjunction with other crosslinking agents, such as hexamine [84] and additional agents like methylene bisacrylamide [82], as it is particularly effective in initiating free-radical polymerization, thereby promoting efficient and uniform network formation.

Various strategies have been employed to promote chemical or physical cross-linking, alongside increasing the number of components used to reticulate hydrogel. In certain cases, radiation approaches have been considered, originating from the early work of Saraydin et al. 2000 [27]. Nonetheless, recent studies have placed diminished emphasis on this method, while interest in procedures employing calcium agents has been increasing, likely due to the emergence of natural materials like alginate [60], pectin [74], or amidated pectin [63].

Regarding the hydrogel forms, the most commonly reported are films, beads, or granules. However, no clear correlation has been observed between the type of compound loaded and the physical form of the hydrogel. Recent innovations have focused on enhancing the spray conditions of conventional herbicides such as dicamba, particularly by achieving an optimal droplet size that minimizes drift and thereby improves efficacy. An example is the development of a hydrogel formulation based on folic acid and zinc nitrate [85], which produced droplets with effective air dispersion and confirmed the potential of this approach for improving spray performance. In the future, such strategies could be adapted to other active compounds, broadening their applicability in sustainable crop protection.

Regarding compound loading, multiple approaches have been identified (see Table S1). The predominant method, reported in 18 studies, is the swelling equilibrium method [28,37,40,49,51,53,56,65,66,67,68,69,70,72,73,74,82,86]. In this approach, the solid hydrogel is immersed in or comes into contact with a compound solution until entrapment occurs. This technique requires low investment and allows the evaluation of the effect of network structure, mesh size, and the affinity between hydrogel material and chemical substances. Another important advantage is its applicability for loading thermally unstable compounds [23]. Additional strategies include encapsulation techniques [11,46,60,63,75,80] or the incorporation of the molecule during hydrogel formation, utilizing an in situ method [27,57,61,62,71,76,79,84,87]. In this latter case, it is important to consider that the compound can be stabilized without compromising its integrity under the temperature, solvent, or pH conditions of fabrication. The advantage of the in situ method lies in its ability to promote stronger interactions between the hydrogel material and the compound. An example is the synthesis of a substance including the herbicide 2,4-dichlorophenoxyacetic acid (2,4-D) and poly[(1-vinyl-2-pyrrolidone)-co-(2-hydroxyethyl methacrylate)] as reported by Pizarro et al. (2008) [76]. The esterification between the hydroxyl groups of the copolymer and the acid chloride groups of the herbicide strengthens these interactions, producing a system in which chemical release is influenced by environmental pH.

**Figure 11:**
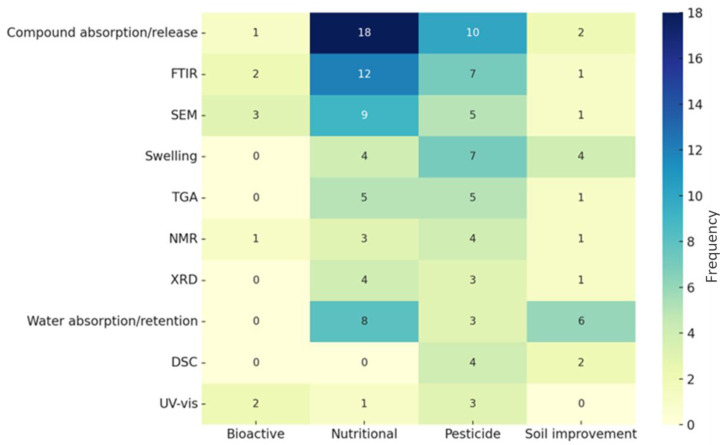
Relationship between commonly used hydrogel characterization techniques and hydrogel categories. Values denote the number of studies reporting each case. Elaborated with the assistance of artificial intelligence (ChatGPT, OpenAI, 2025), based on data and instructions provided by the authors.

Regarding the characterization techniques employed to assess the performance of the hydrogels, as illustrated in Figure 11, the most prevalent methods involve compound absorption and release studies, followed by Fourier Transform Infrared Spectroscopy (FTIR) and swelling tests. Additional techniques such as thermogravimetry analysis (TGA), nuclear magnetic resonance alternatives (NMR), X-ray diffraction (XRD), and differential scanning calorimetry (DSC) provide structural insights into the hydrogels. This prevalence is expected, as the majority of the reviewed studies focus on hydrogel development. Ultraviolet-visible (UV-vis) spectroscopy is also frequently reported as a method to monitor compound release [37,53,63,75,84]. Swelling remains a common technique for assessing hydrogels behavior [11,27,28,38,46,49,56,63,71,79,80,82,83], and the resulting data are often compared with findings from other techniques that examine crosslinking or crystallinity, such as DSC [82].

Microscopic techniques such as scanning electron microscopy (SEM), transmission electron microscopy (TEM), or atomic force microscopy (AFM) are valuable for analyzing material morphology and supporting the evaluation of hydrogel behavior, for example, in studies assessing water absorption capacity [82].

The characterization of hydrogels generally includes testing of absorption, retention, and release of water, particularly in relation to soil moisture retention and the duration of water availability [82]. Some studies emphasize that the integration of specific techniques is essential for understanding the release behavior of active compounds, often using water as a model substance.

Conversely, only seven studied have reported in vivo experiment assessing hydrogel effects on plant growth or seed germination [11,46,61,69,71,79,88] (see Table S1). The plant species studied include varieties of wheat [11,46,88] and lettuce [71,79]. In addition, eight works have been investigated hydrogel-soil interaction tests, either in vivo [46,69,71,88] and ex situ [60,61,79,88], using specific soil types. These investigations focus on degradation and compound release. Despite the diversity of soils considered (see Table S1), natural derived compounds such as fungal biopesticides [70] or garlic oil [46], have been tested in agricultural soil to simulate real application conditions, whereas synthetic pesticides are typically evaluated in soils of defined texture, with attention to their mobility and leaching potential [57,69,71].

The evidence from in vivo and ex situ tests shows that research remains centered on hydrogel design and compound release, with limited attention to plant-level outcomes. Bridging this gap through plant monitoring and field validation will be essential, enabling more effective interdisciplinary collaboration and advancing hydrogel technologies toward sustainable crop protection.

#### 2.2.2. Nutritional Growth Inputs

This category, aligned with ICM strategies, comprises documents addressing compounds supplying essential macro- or micronutrients for plant metabolism, growth, and reproduction. It also encompasses biostimulants that enhance nutrient use efficiency, abiotic stress tolerance, or crop characteristics, regardless of nutrient content [7,12]. A total of 26 documents were categorized in this group, four of which were additionally classified under other categories such as pesticides [28,69], and soil improvement [2,45]. Figure 6 and Table S2 illustrate that the subcategories include fertilizers, which account for the largest number of analyzed documents, followed by biofertilizers and biostimulants.

**Figure 12:**
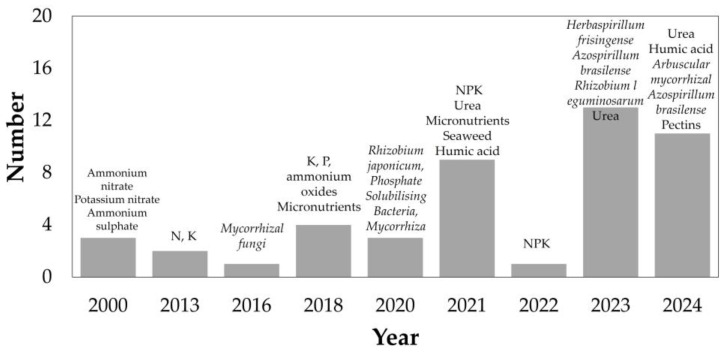
Temporal evolution of nutritional growth inputs reported in the analyzed documents.

The growing variety of fertilizer-related compounds reflects a trend similar to that seen in pesticide applications. Figure 12 shows a significant increase in the diversity of compounds reported in the reviewed documents. Urea remains the most frequently cited substance [20,32,45,50,81,89,90], primarily due to its widespread use as a fertilizer, its high nitrogen content, and low cost. However, nutrient losses and runoff in agricultural systems presents a significant challenge [50].

Seven distinct hydrogel materials have been reported for mitigating urea loss. These include starch-based systems such as starch from rice-cooked wastewater with acrylamide [45], natural rubber latex–cassava starch [90], and acetylated starch–polyacrylamide [81]; natural oil-based coatings with crosslinked PAN/PAAc [50]; PVAl/humic acid hydrogels [20]; and alginate-based formulations, including composites with attapulgite, N-isopropylacrylamide–sodium alginate [89], and sodium alginate–humic acid–NIPAm–AMPS. Additionally, a hydrogel of acrylamide–methylpropanesulfonic acid–poly(ethylene glycol) with sodium alginate and humic acid as a filler has been described [32]. The objectives of these hydrogels are diverse: some aim to create hydrophilic barriers that regulate urea diffusion [50], others focus on establishing strong interactions with urea [45], and some are designed to form complexes through hydrogen bonding or ionic interactions [20]. However, the potential degradation by-products generated from these interactions and their implications for plant growth are not addressed. Overall, the trend indicates that hydrogel systems are evolving—from conventional synthetic designs to more advanced materials incorporating composite structures or smart functionalities, capable of integrating multiple responsive mechanisms, such as combined pH and temperature sensitivity [89].

Table S2 and Figure 12 indicate that humic acid (HA) is the second most frequently cited compound, with its use reported mainly in studies published between 2021 [3] and 2024 [32,34,91]. HA denotes a category of long-chain organic compounds characterized by colloidal properties and complex spatial structures, predominantly originating from plant degradation and fermentation [32]. This substance is widely recognized as an organic fertilizer capable of enhancing plant growth [91]. Researchers have utilized derivatives such as humic acid sodium salt [3] or commercial formulations [32] to develop specific hydrogels. The chemical structure of HA enables hydrogen bonding with hydrogel matrices, thereby improving network stability and while enhancing hydrophilicity, swelling behavior, and compatibility with soil [36].

The incorporation of biological agents, including *Rhizobium* [92,93], *Azospirillum* [52,93], and mycorrhizae [2], has been documented, particularly within bio-based hydrogels made from alginate [52] or pectins [93].

Figure 8 and Table S2 show that a considerable number of hydrogels have been developed, mirroring observations made in the context of pesticides. Figure 9b depicts a specific evolution in the types of polymers utilized for the incorporation of nutritional growth inputs. Between 2000 and 2013, the materials identified predominantly comprised synthetic hydrogels with limited functionality and bio-integration, including acrylamide/crotonic acid [28]. During the subsequent period, as illustrated in Figure 9b and Figure 12, the utilization of commercial hydrogels, including Stockosorb, rose markedly. This material demonstrates benefits in alleviating drought stress in the production of olive plantlets [2]. In this timeframe, biocomposites derived from bacterial nanocellulose were also observed [22]. The latest period examined in this study, extending from 2021 to 2024, underscores the growing application of bio-based hydrogels incorporating components sourced from waste valorization, such as hydrogels derived from rice-cooked wastewater [45].

The trend in nutritional growth inputs indicates a preference for bio-based components in hydrogel production. Various natural polymers have been utilized in these systems, such as sodium alginate [9,32,34,89], starch [45,81,90,94], pectin [93,95], and cellulose [3,9,22,96]. Figure 9b illustrates that this trend has intensified in recent years and is likely to continue. Future developments may focus on multifunctional materials that enhance water retention, facilitate nutrient delivery, and respond to environmental stimuli. An example of such a multifunctional hydrogel is a pH-responsive system based on N-isopropylacrylamide (NIPAm) and 2-acrylamido-2-methyl-1-propanesulfonic acid (AMPS), which exhibits thermal sensitivity and enhanced water absorbency [32].

Further examples of multifunctional hydrogels include composites that integrate natural fillers like attapulgite [89] or biomass sources like pine resins [97]. A notable example is the leather waste–acrylic acid–maleic anhydride composite, which combines water retention, controlled fertilizer release, biodegradability, and heavy metal adsorption—specifically for Cr [III]—while also responding to various environmental parameters such as pH and coexisting ions [32]. This example highlights the incorporation of circular economy principles through the valorization of industrial by-products.

Similarly to observations in documents classified under the pesticide category, a large number of studies on nutritional growth inputs focus—either directly or indirectly—on the analysis of compound release behavior [20,22,32,34,68,81,89,90,91,97,98]. The structural strength of these hydrogels can be influenced by the crosslinking process [20], the incorporation of functional fillers [89], or a combination of both. An illustrative example of this integrated approach is the formulation of composites that combine pine resin polysaccharides with biochar as a filler, enhancing nutrient release and overall material performance [97].

The crosslinking processes utilized in these studies follow a pattern similar to those reported in the pesticide category. Traditional chemical crosslinking systems are widely employed, particularly those involving MBA and APS, most notably in the synthesis of hydrogels from acrylamide derivatives [81,89,97] or acrylic derivatives [35,98]. With the rising adoption of bio-based hydrogels and heightened concerns regarding environmental sustainability, alternative crosslinking strategies have emerged. Ionic crosslinking methods frequently employ divalent or trivalent metal ions, including Ca^2+^ [8,34,52,93], Cu^2+^ [9], and Zn^2+^ [9]. Furthermore, a significant trend is observed in the development of physically crosslinked systems, where interactions such as polymer chain entanglement are crucial, particularly in formulations based on chitosan and starch [94]. Future research is likely to emphasize physical crosslinking mechanisms while reducing reliance on synthetic crosslinkers, initiators, and chemical activators.

For compounds such as humic acid and 3-indoleacetic acid, crosslinking is mainly determined by the interactions between functional groups, particularly hydroxyl and carboxylic acid moieties [3]. Strategies such as coacervation, aldehyde-mediated reactions, and citric-acid-induced crosslinking have shown significant relevance for these substances.

As observed in the case of pesticides, the most employed technique for compound loading in hydrogels for nutritional growth inputs is the swelling equilibrium method, which appears in 46% of the reviewed documents [20,28,32,34,35,45,50,81,89,91,94,96]. The subsequent prevalent technique is the in situ method (27%) [3,8,69,89,95,97,98], followed by encapsulation processes (19%) [22,52,90,93,99]. The frequent application of the swelling equilibrium method may be attributed to its appropriateness for sensitive compounds that are prone to degrade or change during hydrogel synthesis, such as biofertilizers [94]. Even for relatively stable compounds like urea, which can withstand thermal conditions, this method may enhance availability and performance [89]. The selection of a compound loading technique must carefully consider both the chemical stability and availability of the active ingredient under hydrogel fabrication conditions, as previously noted regarding the pesticide category.

Figure 11 illustrates that the main techniques employed to characterize hydrogels for nutritional growth inputs concentrate on compound absorption and release, encompassing release profiles in both water [3,35,50,91,97] and soil [3,20,89,91,97]. As previously discussed regarding pesticide-related hydrogels, structural characterization methods such as FTIR [8,22,35,50,68,81,89,90,91,94,95,98], SEM [9,22,35,50,68,81,89,91,93,94,95,98], and TGA [20,22,45,81,89,91,95,98] are also significantly utilized. Furthermore, evaluations of water absorption and retention capacity are significant for assessing the material’s agricultural performance. The application of these techniques reflects a strong scholarly interest in analyzing or understanding nutrient delivery and availability. The capacity for entrapment of fertilizers, like pesticides, is frequently assessed using UV–visible spectrophotometry [32,90,91,96].

A total of 63 percent of the analyzed documents reports in vivo tests, whereas 40% include germination assays, such as the evaluation of the number of germinated seeds [9,20,45,94,95,96]. The analysis of plant growth encompasses root architectural parameters including length, area, and volume, as well as leaf area measurements [2,9,34,45,68,90,93,94]. Additional trails assess biomass production, including fresh plant or root biomass and dry plant biomass [2,20]. These assays are often complemented by biochemical indicators such as chlorophyll [2,9,94], protein [92], oil content [92], photosynthetic pigment [2], or phenol content [2]. In certain cases, they also provide information on plant responses under water stress conditions [2]. Recent studies have introduced advanced methods, including high-resolution digital imaging technologies, to enable more precise analyses of root architecture [99].

A notable aspect is that 79% of these documents were published between 2023 and 2024, underscoring the growing relevance of the research on hydrogel-plant interactions.

Regarding the types of plants considered, some studies have examined woody species, including olive plantlets (*Olea europaea* L. *cv*.), which are particularly valuable for long-term investigations [2]. Legumes, including soybean [92], mung beans [45,91,95], and peas [45], are commonly employed in early-stage performance testing because of their rapid growth and low experimental costs. Germination assays are frequently performed on grain crops such as maize [34,94] and sorghum [20], whereas evaluations of leafy vegetables typically emphasize traits such as leaf length, root development, and biomass accumulation.

Agricultural soils—including clay loam, sandy phosphorus-deficient, and loamy types—were among those used in the examined studies. Most studies, however, lacked systematic monitoring or characterization soil properties during the experiments. This gap highlights an important avenue for future research, especially to advance understanding of the interactions among soil, hydrogel, and the broader soil–hydrogel–plant system.

#### 2.2.3. Soil Conditioners

Soil conditioners are essential for improving agricultural productivity and mitigating environmental pollution [100,101]. Powlson et al. (2011) [100] identified the primary soil functions that support food production as follows: (1) supporting seed germination, root establishment, and root functionality, including anchorage, water uptake, and nutrient acquisition; (2) acting as a nutrient reservoir; (3) enabling the transport of water and nutrients from both native soil reserves and external inputs; (4) providing a medium for essential nutrient transformations for plant nutrition; (5) supporting diverse microbial and faunal communities that contribute to plant growth and nutrient cycling; and (6) delivering physical support for agricultural operations involving machinery, humans, and animals.

In relation to the analysis of this category, a total of 28 documents were classified. Figure 6 and Table S3 show that the subcategories identified in this review encompass soil bioavailability, microbial activity, mulching, soil structure, and soil water management. Among these, soil water management emerges as the most frequently addressed topic and is supported by the largest number of related documents.

On the other hand, hydrogels have the potential to modify and enhance the fluid dynamics of the soil liquid phase by (1) increasing water-holding capacity, (2) improving water flow characteristics, and (3) enhancing the mechanical performance of soils under diverse environmental stress conditions [102,103].

This category reveals extensive use of diverse hydrogel systems (see Figure 8 and Figure 9c, and Table S3). Synthetic polymers, particularly polyacrylamide-based hydrogels [45,47,104,105], include innovative examples such as poly(acrylamide-co-acrylate) derived from acrylamide processing residues [104]. Commercial products like Stockosorb 660 are also reported [2,42]. Beyond these, nanocomposite formulations, such as poly(acrylic acid-co-acrylamide)/AlZnFe_2_O_4_–potassium humate [47], and hybrid systems, including poly(acrylic acid)-graft-agar/gum Arabic [106] and chitosan/potato starch blends [10], demonstrate efforts to integrate functionality. At the same time, biopolymer-based hydrogels, often incorporating natural polymers or agricultural waste [10], highlight a clear shift toward environmentally sustainable systems consistent with circular economy principles. As further shown in Figure 9c, several hydrogels can be classified as hybrid, smart [106], or multifunctional systems within the previously defined categories. Multifunctional hydrogels play an important role in soil water management, structural improvement, and the stimulation of microbial activity [33].

On the other hand, as reported in Table S3, bio-based hydrogels are predominantly associated with soil structure [33,44,54,103,107,108] and soil water management [36,45,55,106,109,110,111].

Moreover, these hydrogels allow regulated water release in response to external stimuli such as temperature, pH, light, and enzymatic activity [79]. As shown in Figure 9c, since 2017 there has been a marked increase in the development of bio-based, hybrid, smart, and multifunctional hydrogels, reflecting sustained innovation directed toward sustainable agricultural applications. The application of nanocomposite hydrogels can be traced to the period between 2009 and 2012 [47].

**Figure 13:**
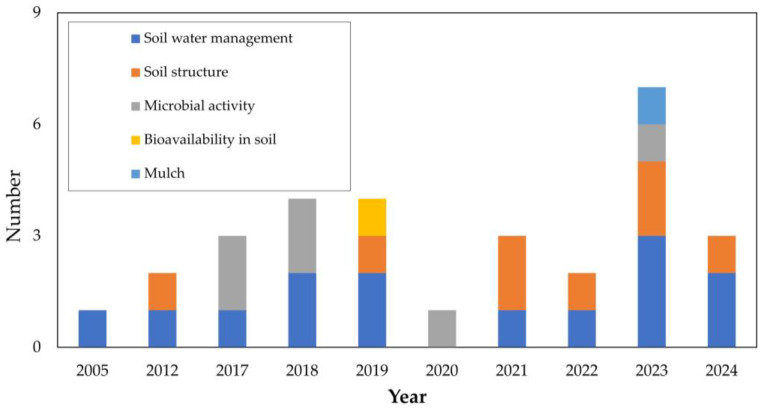
Temporal evolution of soil improvements reported in the analyzed documents.

Figure 13 highlights that soil water management consistently emerges as a central theme among authors. Soil structure has gained increasing relevance since 2012, with a particularly strong emphasis after 2021. Microbial activity has been addressed intermittently, appearing in works from 2017 [1,112], 2018 [2,10], 2020 [113], and 2023 [93]. Conversely, the subcategories of bioavailability in soil and mulch remain rarely examined, despite their potential importance for ICM strategies.

Regarding the crosslinking process, ionic crosslinking—using divalent ions such as Ca^2+^ or Cu^2+^—is frequently employed, particularly in applications related to soil structure and microbial activity [93,113]. In contrast, commercial formulations or thermally crosslinked hydrogels are more common in studies focused on soil water retention. As in other categories, chemical crosslinking with MBA remains the dominant approach, especially for soil water management, due to its proven effectiveness in improving water-holding capacity [36,104,106,109].

Hydrogel presentation typically includes films and different types of granulates, such as beads, consistent with other categories. A liquid formulation was also identified, noted for its improved mixing capacity with the soil [103].

Figure 11 shows that the main hydrogel characterization methods in this category are water absorption and retention [1,10,47,48,104,107,109,110,113,114], followed by swelling analysis [10,31,55,106,107,109,112]. In addition, several studies report techniques related to thermal stability and degradation, including TGA, DSC, and related methods [10,33,45,55,107,108].

A total of 19 studies were identified that evaluated hydrogel under soil-based conditions. The soils tested were mainly characterized by low fertility, poor structure, and limited water retention, including sandy loam [1,2,36,42,47,54,103,113], saline-alkali [33], and coarse-textured soils [44,54,103,112]. This pattern underscores the importance of testing hydrogels for soil water management, a critical agricultural application. Bio-based hydrogels were also studied in cultivated soils [45,115], garden soils [110], and natural soils such as forest soil [111], reflecting attempts to design ecologically relevant testing framework across diverse conditions.

In vivo assays were documented in 13 documents, four of which evaluated germination [47,55,111,114]. Table S3 indicates that between 2012 and 2018, studies mainly focused on staple crops such as wheat [47], maize [116], and olive [2,42]. In later years, a wider range of species has been tested, including *Lactuca sativa* [115], tomatoes [48,111], potatoes [104], legumes such as *Pisum sativum* [110] and *Vigna radiata* [45,110], leafy vegetables from the *Brassica* [114] and *Capsicum* [114], and several weed species [114]. This trend demonstrates a growing interest in the broader agroecological role of hydrogel.

In relation to germination, it is also important to highlight that the incorporation of hydrogels can also alter soil porosity and permeability, thereby influencing seedling performance [47,55].

**Figure 14:**
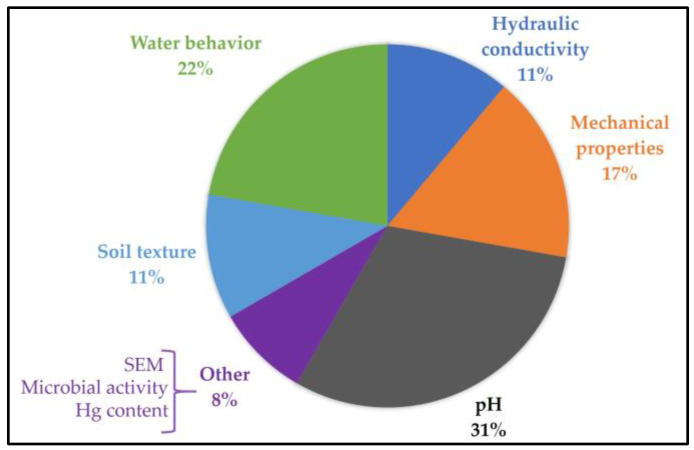
Main soil characterization parameters reported in the reviewed studies, expressed as percentage distribution.

Finally, at least 30 distinct have been identified for evaluating soil-hydrogel interactions, categorized in Figure 14 and detailed in Table S3. Despite this variety, two key aspects consistently emerge as fundamental in study design: soil pH and water dynamics.

#### 2.2.4. Bioactive

Bioactive substances contribute to ICM practices by functioning as organic insecticides, growth regulators, and disease inhibitors, thereby promoting plant growth and improving soil health [117]. These compounds are typically categorized into phenolic compounds, non-phenolic compounds, and pigments [118]. In addition, bioactives can act as biochemical probes, serving as model systems for studying biochemical processes and controlled release behavior [119].

Only three documents met the criteria for this category, as reported in Table S4. These primarily examined compounds employed as model systems to track hydrogel-controlled release. Notably, phenolic molecules such as phloroglucinol (1,3,5-benzenetriol) were identified as effective probes for evaluating the performance of hydrogels synthesized from methyl-esterified pectin and humic substances derived from composted biomass [120]. Furthermore, two investigations focused on highly porous, tunable biohydrogels formed from metal ions and organic ligands, specifically amino acid-enriched collagen–starch matrices [58,121]. These systems demonstrate direct interactions with root and leaf tissues, enhancing plant metabolism, tissue development, and overall plant growth.

Although the number of documents in this category is limited (Table S4), the studies reveal a consistent reliance on bio-based materials. For instance, ionic crosslinking with Ca^2+^ was applied due to the natural presence of pectins in hydrogel matrices [120], while polyurethane was incorporated into semi-interpenetrating polymer networks (semi-IPNs) [58,121]. Compound loading strategies included swelling equilibrium and encapsulation methods, as observed in earlier categories. The analytical approaches used to characterize both hydrogels and plant responses were consistent with those previously described.

Importantly, in vivo evaluations were performed with the same plant species, tomato (*Solanum lycopersicum*), in both studies [58,121]. The preference for tomato is likely linked to its widespread role as a model organism in plant science, particularly for investigations of plant defense mechanisms against pathogens affecting fruit quality [122]. This relevance is reinforced by the availability of extensive genetic and genomic resources supporting its use in experimental frameworks [123].

### 2.3. Opportunities for Future Research

This study illustrates how diverse types of hydrogels can be effectively integrated with ICM strategies to address persistent challenges in the agricultural sector. The development of hydrogels for applications such as pesticides, nutritional growth inputs, soil conditioners, and bioactive compounds represents a dynamic and expanding field of global research. Particular emphasis is observed in initiatives focused on bio-based multifunctional materials, the valorization of industrial by-products, and the reuse of post-consumer waste. These approaches enhance hydrogel performance while adhering to the principles of circular economy and green chemistry. Based on this analysis, several key opportunities for future research are identified:

- Conduct detailed investigations of hydrogel-soil interactions, with special emphasis on soil microbiota and plant–microbe dynamics.
- Expand field-based assessments in practical agricultural environments [2]. While the number of such studies has increased, further research is particularly needed in underexplored contexts, such as flooded soils.
- Examine the potential toxicity of hydrogels in soil and aquatic systems. This remains insufficiently studied, as highlighted by Kolya et al. 2023 [45], despite the likelihood of large-scale applications.
- Evaluate hydrogels performance under a wide range of environmental conditions. Research has largely prioritized drought stress, but greater attention is needed to conditions such as extreme moisture, atmospheric variability, and non-traditional systems, including soilless, urban, and peri-urban agriculture.
- Integrate regulatory aspects into hydrogel research. Future studies must address regulatory frameworks and toxicity assessments, as their absence represents a critical barrier to the safe, sustainable, and large-scale agricultural application of hydrogels.
- Despite the identification of bio-based hydrogels in this study, including those derived from agro-industrial sources, further exploration of alternatives from food waste as well as urban and industrial residues remains essential.
- None of the analyzed studies address the environmental impact of the developed hydrogels. This gap highlights a key opportunity for future research to integrate green engineering principles with environmental systems analysis, including carbon neutrality, life cycle assessment, and water footprint evaluation.
- The reviewed documents did not report the implementation of strategies such as machine learning models or simulation tools for predicting hydrogel behavior. Incorporating these approaches could improve resource utilization and leverage the expanding corpus of scientific and technical knowledge.
- Other identified opportunities involve long-term evaluation, particularly through integration of multiple ICM strategies that combine pesticides, biostimulants, biofertilizers, and soil conditioners [7] to enhance crop production, potentially complemented by mulch application [114].
- Incorporating emerging technologies, such as additive manufacturing or rapid prototyping, which rely on layer-by-layer fabrication technology [124], could support the development of complex structures. Multifunctional hydrogel may particularly benefit from these methods, which offer greater flexibility and reduced material waste [124].
- Only one study was identified that investigated the use of hydrogel as mulch. The combination of hydrogels with film-based alternatives represents a promising direction for developing multifunctional materials that integrate soil protection, water management, and compound release, particularly relevant to small-scale food production.
- This study demonstrates a growing interest in hydrogel applications; however, additional research is essential, especially for the transition from laboratory prototypes to real-scale applications. Such efforts should foster broader engagement from researchers across industrial sectors.
- Various researchers have highlighted the need to investigate alternatives to the conventional soil integration of hydrogels, such as packaging them in bags made from hygienic mask wastes [110], while also exploring techniques for reprocessing or refilling the hydrogel [23]. These methods may reduce reliance on raw materials and continuously strengthen the circular economy.
- Variations in the crosslinking process were also identified, reflecting efforts to lower the environmental impact compared with conventional methods. These trends are likely to intensify in the future, particularly for hydrogels used as chemical release devices. However, it is crucial to examine the potential by-products arising from hydrogel-compound interactions, as well as any resulting contaminants and their impacts on plants or soil.

## 3. Conclusions

This work presents a concise overview of the current development of hydrogels in agricultural practices, framed within ICM strategies. This analysis was conducted through a synthesis of bibliometric analysis and PRISMA methodology. The approach included macro- and micro-level analyses that identified the worldwide significance of the topic, identified the regions of the greatest relevance, and examined key research domains through the analysis of the author’s keywords and selected publications. The trend has been increasing steadily, especially during the second decade of the twenty-first century, and appears promising for the coming years. Contributions from authors worldwide have been documented, reaffirming the topic’s significance. Recent developments highlight the importance of green and sustainable agriculture, together with the expanded use of agro-waste residues in hydrogels, offering opportunities for implementing a circular economy. Guided by the ICM strategies, the selected documents were categorized into four groups: (1) pesticides, (2) nutritional growth inputs, (3) soil conditioners, and (4) bioactives. Each category was examined for patterns, with particular emphasis on identifying materials for future study in this domain.

The significance of hydrogels within the ICM framework is clear, as is the imperative to further develop advanced hydrogels from bio-based components that can function as multifunctional materials, broadening their agricultural applications. Thus, materials such as multilayer systems, nanocomposites, or biocomposites may serve as viable alternatives. Furthermore, the potential for enhancing interactions through the incorporation of the specific component within the hydrogel should be examined to adjust the crosslinking degree. However, it is essential to investigate the possible formation of by-products that may affect plants and soil during application.

A wide range of characterization methodologies were identified, most of which are based on hydrogel behavior. This underscores the need to strengthen research on hydrogel interactions with both plants and soil. Overall, this work highlights the necessity of fostering strong collaboration between industry and academia, supported by international cooperation, to advance these developments and achieve meaningful impacts in addressing global food production challenges under climate change.

## 4. Materials and Methods

This study was carried out using a rigorous process that integrated the Preferred Reporting Items for Systematic Reviews and Meta-Analyses (PRISMA) methodology and bibliometric analysis [125,126]. To minimize bias and ensure reproducibility, the PRISMA technique was selected [125,126].

**Figure 15:**
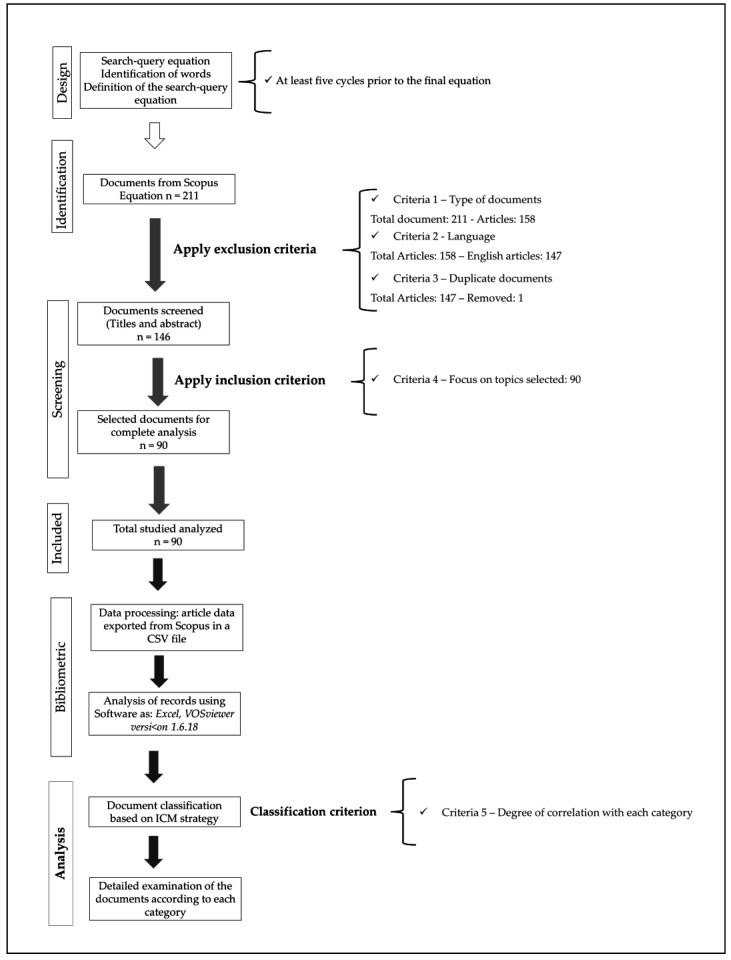
Scheme to conduct bibliometric and systematic analyses.

Figure 15 summarizes the methodology applied in this study. The first step was to define the search-query equation. A minimum of five iterations were conducted before generating the final version. This procedure ensured a broader retrieval of relevant documents.

During this iterative process, it was possible to identify terms corresponding to different substances used as agrochemicals categories within ICM, including fertilizers, plant protection agents, and biostimulants. Given the diversity of biostimulants types and the challenges in establishing a comprehensive definition [7], this work adopted the main categories proposed by Du Jardin (2015) [7].

The final search-query was defined as [humic OR fulvic OR “amino-acid*” OR peptid* OR “protein hydrolys*” OR seaweed OR algae* OR “inorganic salt*” OR fungi* OR mycorrhiza OR bacterial* OR herbicid* OR “weed control” OR bioherbicid* OR biofertilizer* OR biostimulant*) AND [hydrogel*) AND [agricultur*) AND NOT [medicin* or scaffold* OR biosen* OR biomedical OR “solar steam” OR “stem cel*”). The Boolean operator AND NOT was used to exclude unrelated publications, particularly those addressing biological, medicine, sensors, or toxicological applications where hydrogels are also employed. To maximize retrieval, the operator asterisk (*) was used to capture variations in relevant terms.

The next step was to search for the documents in a database. In this case, the Scopus database was selected because it contains a comprehensive record set [127,128] and offers reliable access to information. However, this choice also represents a limitation, as non-database sources include all relevant publications [129]. Nonetheless, Scopus is widely used and facilitates accessible and reproducible searches.

As in previous steps, the equations were applied to the TITLE-ABS-KEY fields in Scopus. All documents published between 1999 and 31 December 2024, were retrieved. As shown Figure 15, a total of 211 records were initially obtained. Because this study focused on research articles, reviews and other document types were excluded. After applying this criterion, 158 articles remained (Figure 15). Additional exclusion criteria include language, English-only and duplicate entries, ensuring accessibility and consistency. Following these steps, the total number of articles was reduced to 146.

As indicated in Figure 15, the next step involved an extensive review of titles and abstracts. At this stage, the inclusion criterion was limited to documents explicitly addressing the use of hydrogel in soil health or quality, pest control, or crop nutrition. Documents were excluded if they focused on topics such as filtration, fruit packaging, fruit protection, sensor development, water pollution control, reviews not identified earlier, agricultural practices without hydrogel, hydrogel processing or characterization, genetics, medical applications, or biotechnology. There are fifty documents in all that have been deleted. A total of 50 documents were excluded. Ultimately, 90 articles were retained for bibliometric and in-depth analysis.

The Scopus data for the final set of documents was exported as comma-separated values (csv) files. Data processing and visualization were conducted using VOSviewer version 1.6.18, Microsoft Excel^®^, and SankeyMATIC, and were additionally supported by artificial intelligence tools (ChatGPT, OpenAI, 2025), based on data and instructions provided by the authors.

As noted in the preceding section, a macro- and micro-level bibliometric analysis was applied to identify factors such as the most active state, notable institutions, and leading authors. The visualization of data was particularly valuable for revealing collaborative networks of the co-authors, while the analysis of prevalent author keywords illustrated the evolution of research topics over time, and allowed the identification of clusters and thematic areas, as reported by other authors [26,130].

In addition, the final selected articles were thoroughly reviewed using the methodology established in prior studies [126] to determinate the most important components of the topic categories. The selected documents underwent systematic review, cataloging, and analysis across at least three work iterations. Microsoft Excel^®^ was used to create association graphs for categories, and also it was used to create diagrams free software such as SankeyMATIC https://sankeymatic.com/ (accessed on 20 July 2025).

## Data Availability

The data reported are available by contacting the corresponding author.

## Supplementary Materials

The following supporting information can be downloaded at: https://www.mdpi.com/article/10.3390/gels11090731/s1, Table S1: Documents classified in the Pesticides category; Table S2: Documents classified in the Nutritional growth inputs category; Table S3: Documents classified in the Soil conditioners category.; Table S4: Documents classified in the Bioactive category.
