# Supplementary material for: Analysis of the Growth of Hydrogel Applications in Agriculture: A Review
Source: Gels. 2025 Sep 11;11(9):731. doi: 10.3390/gels11090731 (PMC12469933; doi:10.3390/gels11090731)
Supplement: Supplementary file 1 [file gels-11-00731-s001.zip › Table S1.pdf]

Table S1. Documents classified in the Pesticides category.

Hydrogel materials	Type of compound	Compound	Hydrogel preparation or crosslinking process	Compound loading	Characterization of materials	Plant parameter		Soil parameter			References
						Plant	Germination or growth	Soil type	Soil/Hydrogel	Soil analysis	
Acrylamide/crotonic acid	Herbicide	Dalapon (sodium 2,2-dichloropropionate)	Crosslinking process using ethylene glycol dimethacrylate and 1,4-butanediol dimethacrylate	Swelling equilibrium method	Swelling; water absorption	---	---	---	---	---	Karadağ et al., 2000 [28]
Poly(acrylamide/itaconic acid)	Herbicide	Sodium 2,2 dichloropropionate - Commercial product: Dowpon	Radiation polymerization for crosslinking process	*In situ* method	Swelling; diffusion	---	---	---	---	---	Saraydin et al., 2000 [27]
Polyacrylamide/poly(ethylene oxide)	Herbicide	Atrazine	Irradiation electron beam for crosslinking process	Swelling equilibrium method	XRD; TGA; DSC; SEM; 1HNMR; water absorbency; compound release; gel determination	---	---	---	---	---	Abd El-Rehim et al., 2005 [65]
Poly[(1-vinyl-2-pyrrolidone)-co-(2-hydroxyethyl methacrylate)]	Herbicide	2,4-dichlorophenoxy acetic acid (2,4-D)	Radiation polymerization for crosslinking process, and BPO as the initiator	*In situ* method	FTIR; H NMR; GPC; TGA	---	---	---	---	---	Pizzaro et al., 2008 [77]
Psyllium/acrylic acid	Fungicide	Copper sulfate	Gamma irradiation for crosslinking process	Swelling equilibrium method	FTIR; XRD; UV VIS	---	---	---	---	---	Kumar and Kaith, 2010 [37]

Starch/ethylene glycol-co-methacrylic acid	Herbicide Fungicide Insecticide	Fluometuron (herbicide); thiophanate methyl (fungicide); trifluralin (insecticide)	Gamma irradiation for crosslinking process	Swelling equilibrium method	UV VIS; DSC; SEM; FTIR	---	---	---	---	---	Abd El-Mohdy et al., 2011 [73]
Alginate/agar/starch	Biofungicide	Fungal biopesticides: *Nomuraea rileyi*	Not reported	Swelling equilibrium method	Evaluation of biocontrol potential against *Spodoptera litura*	---	---	Fertile loam soil	Biocontrol against *Spodoptera litura*	---	Namasivayam and Vidyasankar, 2014 [70]
Attapulgite clay/polyacrylamide acrylic composite	Herbicide	Atrazine; alachlor; tribenuron-methyl	Unspecified process	Swelling equilibrium method	ESEM; Fractal dimension	---	---	---	---	---	Liao, 2014 [66]
Agar/starch	Herbicide	Atrazine	Crosslinking process using MBA and APS as the initiator	Swelling equilibrium method	FTIR; XRD; SEM	---	---	---	---	---	Singh et al., 2015 [51]
Guar gum-graft-poly(acrylate)	Fungicide	*Trichoderma harzianum*	Radical grafting polymerization; Crosslinking process using MBA and persulphate as the initiator	Immobilization of biological individuals	FTIR; C NMR; SEM; CHNS; In vitro bioefficacy of material against *P. aphanidermatum*	---	---	---	---	---	Chandrika et al., 2016 [84]
Guar gum-cross linked-poly(itaconic acid)	Biological control	---	Crosslinking process using hexamine and APS as a initiator	*In situ* method	FTIR; TGA; SEM; UV VIS; antibacterial activity; swelling; water retention in different soils; antibacterial activity to *Staphylococcus aureus* (MTCC 737)	---	---	Silt and clay	---	---	Sharma et al., 2017 [85]

Guar gum-grafted-crosslinked-polyacrylate/bentonite clay hydrogel composite	Herbicide	Imazethapyr	Crosslinking process using MBA and APS as the initiator	Hydrogel powder was added to compound solution	FTIR; SEM; TEM; HPLC; Release of imazethapyr in water	Garlic	---	Alluvium and sandy-loam soil	Effect on weeds	---	Kumar et al., 2017 [69]
Commercial reference: Aquasorb based on polyacrylamide and acrylic gel	Fungicide	Pathogenic microflora of potato *Oomycete phytophthora* and *Enterobacterium Pectobacterium atrosepticum*	Not mentioned	Not mentioned	Microbiological analysis and estimation of antimicrobial effective concentrations	---	---	---	---	---	Smagin et al., 2018 [78]
Guar gum-cross linked-soya lecithin nanohydrogel	Fungicide	Removal thiophanate methyl	Crosslinking process using MBA and microwave treatment	Swelling equilibrium method	FTIR; XRD; TEM; SEM	---	---	---	---	---	Sharma et al., 2018 [43]
Potassium acrylate with protein hydrolysate filler composite	Pathogen control	Not mentioned	Crosslinking process using MBA and divinylbenzene	---	Spikelets number; spike length: spike weight; grain weight	Soft wheat, Trizo, k-6498 (*Triticum aestivum L.*); Triticale Dua, k-828 i (× Triticosecale)	---	19 indicators of wheat productivity. Some of them are morphological features; wheat yield in the earing; flowering; ontogenesis phases; flag and pre-flag leaves area; grains weight of spike per one plant; grains weight per spike	Not mentioned	---	Kolesnikov et al., 2021 [89]

Chitosan/acrylic acid with copper oxide nanocomposite	Fungicide	Copper oxide nanoparticles	Crosslinking process using MBA and APS as the initiator	*In situ* method	FTIR; hydrodynamic diameter; TEM; SEM-EDS; Swelling test; Cu release in water; Cu release in soil; resistant to *Fusarium oxysporum f. sp.*	Lettuce (*Lactuca sativa*)	Fresh roots biomass; fresh shoot biomass	---	Fresh roots biomass; fresh shoot biomass	---	Shang et al., 2021 [71]
Based on folic acid and zinc nitrate	Herbicide	Dicamba	Not mentioned	Spraying process	SEM; rheology; performance of droplet drift	---	---	---	---	---	Song et al., 2021 [86]
CMC/poly(methacrylic acid)-co-polyacrylamide/clinoptilolite nanocomposite	Herbicide	Diquat dibromide monohydrate. Commercial product: Diquat	Free-radical polymerization Crosslinking process using MBA and APS as the initiator	Swelling equilibrium method	SEM; EDX; XRD; DSC; swelling at different pH; compound sorption; compound desorption	---	---	---	---	---	Tanaka et al., 2021 [38]
Carboxymethyl-chitosan	Fungicide	Prothioconazole	Emulsion-gelation Crosslinking process using metal ions as Cu^{2+}, Mn^{2+}, and Zn^{2+}	Encapsulation process	SEM; EDS; FTIR; XRD; HPLC; Loading content and encapsulation efficiency; fungicide release; swelling; In vitro release; behavior against G. graminis,	Seeds of wheat variety named "Nongda 211"	Height of different plant parts; fresh weight of plant	---	---	---	Xu et al., 2021 [11]
Poly(γ-glutamic acid)/gelatin	Biological detoxification	Genetically engineered version of the zearalenone hydrolase (rdZHD101) enzyme	Not reported	*In situ* method	HPLC; SEM; thermostability; pH stability; activity and stability of immobilized	---	---	---	---	---	Fu et al., 2021 [88]

Starch/alginate/polyacrylamide	Fungicide	Thiophanate-methyl	Crosslinking process using MBA and APS as the initiator	Encapsulation process	SEM; AFM; FTIR; XRD; 13C NMR; TGA; DSC; swelling test: fungicide release	---	---	---	---	---	Singh et al., 2022 [81]
Starch-chitosan	Herbicide	Atrazine	Crosslinking process using glyoxal	Swelling equilibrium method	FTIR; SEM; TGA; DSC	---	---	---	---	---	Supare et al., 2022 [67]
Alginate-g-poly(NIPAm-co-DEAAm) graft copolymer	Herbicide	Glyphosate	Ion crosslinking	Swelling equilibrium method	FTIR; SEM; XRD; TGA; Zeta potential; rheology; compound release	---	---	---	---	---	Zheng et al., 2022 [68]
Sodium alginate (AL) and CMC	Disinfectant	Glycoalkaloids extracted from tomato and potato leaves	Crosslinking by the use of Ca^{2+} ions	Encapsulation process	ATR-FTIR; SEM; TGA; DSC; HPLC-MS; encapsulation efficiency; compound release; microbial test using same species of the soil analysis	---	---	Agricultural soil	Mixed with soil	Aspergillus Analysis of presence of *Aspergillus brasiliensis, Aspergillus fumigatus, Fusarium oxysporum, Tricophyton mentagrophytes and Candida albicans* as the mycotic species; *Pseudomonas aeruginosa, Enterococcus faecalis, and Escherichia coli* as representative bacteria.	Clemente et al., 2023 [60]

Alginate-g-poly(NIPAm-co-DEAAm) graft copolymer	Fungicide	Thiophanate methyl	Crosslinking process by methylene bisacrylamide and APS as the initiator	Swelling equilibrium method	FTIR; XRD; SEM; AFM; 13C NMR; swelling, compound release; water retained by soil	---	---	---	---	---	Singh et al., 2023 [83]
Gellan gum	Herbicide	Glufosinate	Crosslinking process using calcium chloride solution	*In situ* method	FTIR; CHNS; TGA	Crabgrass weeds	Visual inspection	Non-specified	---	---	Azahari et al., 2023 [61]
Core: MIL-101(Fe^{3+}) Nanoparticles<br>Material: Metal–organic framework (MOF) made from $FeCl_3$ and 2-aminoterephthalic acid.<br><br>Structure: Mesoporous crystalline cages with high surface area (1822 m^2/g).<br><br>Function: Serves as the carrier for encapsulating paraquat (PQ), a widely used herbicide.	Herbicide	Paraquat	Crosslinking process using calcium chloride	Swelling equilibrium method	SEM; XRD; TGA; Zeta potential; BET; EDX; contact angle; DLS; EDX; UV stability (Photodegradation); compound release	---	---	---	---	---	Dong et al., 2023 [53]
Carboxymethyl cellulose/cellulose nanofibers nanocomposites	Fungicide	Garlic oil	Ionic gelation	Encapsulation process	FTIR; TGA; SEM; swelling; antifungal activity; water holding and retention capacities of soil,	Wheat seeds	Germination process	Sandy soil	Performance of soil	---	Frühauf et al., 2023 [72]
β-cyclodextrin-polyacrylamide crystalline porous materials	Fungicide	Paclobutrazol; hexaconazole; flusilazole; propiconazole; tebuconazole	Crosslinking process using β-cyclodextrin-methacrylate	Swelling equilibrium method	SEM; FTIR; TGA; XPS	---	---	---	---	---	Wang et al., 2023 [87]
Carboxymethyl chitosan/sodium alginate	Fungicide	Hymexazol	Reaction mixture Non-covalent crosslinking	*In situ* method	SEM; rheology; HPLC	---	---	---	---	---	Xu et al., 2023 [62]

Methylcellulose or chitosan with zeolite, supported by a poly(methacrylic acid)–co-polyacrylamide nanocomposite	Herbicide	Diquat	Crosslinking process using MBA and potassium persulfate as the initiator	Swelling equilibrium method	FTIR; SEM; XRD; swelling test; compound sorption and desorption	---	---	---	---	---	Tanaka et al., 2023 [49]
Ca- alginate graft copolymer of poly(N-isopropyl acryla-mide-co-N,N-diethylacrylamide)	Herbicide	2,4-dichlorophenoxy acetic acid	Crosslinking promoted by LG-MA	*In situ* method	FTIR; NMR; SEM; XPS; HPLC; swelling; agrochemical content; compound release; absorbance capacity of heavy metal ions	Lettuce seeds	Growth rate of lettuce seeds; plant height; root length	Filter paper	---	---	Zheng et al., 2023 [80]
Agar/gelatin	Herbicide	Linuron	Crosslink process using MBA and APS as the initiator	*In situ* method	FTIR; XRD; TGA; SEM; AFM; 13C NMR; compound release	---	---	Sandy loam soil	---	Soil water holding capacity	Singh et al., 2024 [57]
Composites based on sodium alginate/activated carbon	Herbicide	2,4-dichlophenoxyac etic acid	Crosslinking process using calcium chloride	Swelling equilibrium method	DRX; FTIR; SEM; EDX; BET; Zeta potential; dynamic adsorption	---	---	---	---	---	Naboulsi et al., 2024 [74]
Chitosan–pectins	Herbicide	Plicoram	Crosslinking process using calcium chloride, and was during synthesis	Swelling equilibrium method	FTIR; SEM; mechanical testing; swelling tests; compound release	---	---	Clay loam soil	---	---	Adi Sulianto et al., 2024 [75]
Alginate/amidated pectin	Biological control	*Trichoderma spp.*	Crosslinking process using calcium gluconate	Encapsulati on process	SEM; particle size; swelling; Viability Test with Trichoderma koningiopsis	---	---	---	---	---	Cruz-Barrera et al., 2024 [63]

Commercial nanocellulose crystals	Fungicide	Azoxystrobin	Chemical modification of cellulose	Encapsulati on process	UV VIS; FTIR; SEM; TEM; zeta potential; contact angle; antifungal activity; efficacy of encapsulated prototypes against rice sheath blight	---	---	---	---	---	You et al., 2024 [76]
Methylcellulose or chitosan with zeolite, supported by a poly(methacrylic acid)–co-polyacrylamide nanocomposite	Herbicide	Paraquat; Difenzoquat (DFZ)	Crosslinking process using MBA and APSte as the initiator	Swelling equilibrium method	Swelling, TGA; apparent viscosity; compound sorption; compound desorption	---	---	---	---	---	Tanaka et al., 2024 [56]
