# Supplementary material for: Analysis of the Growth of Hydrogel Applications in Agriculture: A Review
Source: Gels. 2025 Sep 11;11(9):731. doi: 10.3390/gels11090731 (PMC12469933; doi:10.3390/gels11090731)
Supplement: Supplementary file 1 [file gels-11-00731-s001.zip › Table S2.pdf]

Table S2. Documents classified in the Nutritional growth inputs category.

Hydrogel materials	Type of compound	Compound	Hydrogel preparation or crosslinking process	Compound loading	Characterization of materials	Plant parameter		Soil parameter			References
						Plant	Germination or growth	Soil type	Soil/Hydrogel	Soil analysis	
Acrylamide/crotonic acid	Fertilizer	Ammonium nitrate, potassium nitrate and ammonium sulphate used	Crosslinking process using ethylene glycol dimethacrylate and 1,4-butanediol dimethacrylate	Swelling equilibrium method	Swelling; water absorption	---	---	---	---	---	Karadağ et al., 2000 [28]
Acrylic acid/humic acid	Fertilizer	Nitrogen fertilizer; phosphate fertilizer	Crosslinking with MBA and APS as the initiator	Swelling equilibrium method	FTIR; SEM; water absorption; water desorption; compound absorption; compound desorption	---	---	---	---	---	Gao et al., 2013 [35]
Commercial material: Stockosorb 660-medium	Biostimulant	Mycorrhizal fungi inoculation	---	Adding to plant	---	Olive plantlets (*O. europaea L. cv.*)	Stomata density; trichome densities; total phenols; leaf area; height plant; diameter of the stem; number of leaves; leaf relative water content; plan dry biomass, photosynthetic pigment; phenol content; chlorophyll	Sandy P-poor soil	Mixed with soil	Soil moisture	M'barki et al., 2018 [2]
Bacterial cellulose–poly(AA-co-MBA) nanocomposite	Fertilizer	Potassium, phosphorus, ammonium oxides and micronutrients	Crosslinking using MBA and potassium persulphate as the initiator	Encapsulation process	FTIR; TGA; SEM; XRD; rheology; profile release; swelling	---	---	---	---	---	Zaharia et al., 2018 [22]

Polymer matrix–nanoclay composite	Biofertilizer	Inoculated *Rhizobium japonicum, Phosphate Solubilizing Bacteria, Mycorrhiza*	Not mentioned	Located separately in the soil	---	Soybean (*Glycine max L. Merrill*)	Plant height; number of branches; protein, oil content	Clay loam	Located separately in the soil	---	Kumar et al., 2020 [93]
Dual-layer coating with natural oils and crosslinked PAN/PAAc	Fertilizer	Urea	Polymerization reaction using gamma (γ)-ray irradiation	Swelling equilibrium method	FTIR; SEM: urease-colorimetric assay; water-retention; in vitro antibacterial assay of hydrogel-coated urea granules against PGPR	---	---	---	---	---	Ghobashy et al., 2021 [50]
Leather waste–acrylic acid–maleic anhydride composite	Biofertilizer	Collagen-nitrogen and potassium	Crosslinking using MBA and APS as the initiator	*In situ* method	SEM; EDX; FTIR; TGA; swelling; release nitrogen; release K+; biodegradation in soil; Cr (III) adsorption the model pollutant	---	---	Soil field	---	---	Hu et al., 2021 [99]
Gellan gum/seaweed	Biofertilizer	Seaweed	Crosslinking process using calcium chloride	*In situ* method	FTIR; CHNS; VPSEM; swelling; degradation test by lose water	---	---	---	---	---	Jaafar et al., 2021 [8]
Seed coating: based on NPK solution and different components as sodium alginate, gum Arabic, amino acid solution or carboxymethyl cellulose	Fertilizer	NPK; source of fertilizer micronutrients: Cu(II), Mn(II), Zn(II))	Crosslinking using (Cu(II), Mn(II), Zn(II)) and an AA solutions	Coating of seed using material based on NPK and different components	Optical microscopy; SEM; EDX; NMR; extraction tests; amino acid composition	Cucumbers (Cornichon de Paris)	Germination test; Plant growing evaluation: Chlorophyll content; stem length; root area; root length; root volume	---	---	---	Skrzypczak et al., 2021 [9]

Cellulose–clay–humic acid composite	Biostimulant	Humic acid	Crosslinking by the presence of alcoholic, aldehyde, and carboxylic acid groups	*In situ* method	Degradation tests (water and soil); swelling	---	---	Coastal soil; beech forest soil; agricultural soil	Mixed with soil	---	Turioni et al., 2021 [3]
Biochar-pine resin/acrylamide composites	Fertilizer	NPK	Crosslinking using MBA and APS as the initiator	*In situ* method	Water retention; bulk density; porosity; water absorption; compound release; compound release in soil; biodegradation (soil)	---	---	Soil from research farm	Mixed with soil	---	Das et al., 2022 [98]
LG-MA–based pH-responsive hydrogel with DCP-MA or IAC-MA	Biostimulant	3-indoleacetic acid	Crosslinking promoted by LG-MA	*In situ* method	FTIR; NMR; SEM; XPS; HPLC; swelling; agrochemical content; compound release; absorbance capacity of heavy metal ions	Lettuce seeds	Growth rate of lettuce seeds; plant height; root length	Filter paper	---	---	Zheng et al., 2022 [68]
Amidated pectin	Biofertilizer	*Herbaspirillum frisingense AP21; Azospirillum brasilense D7; Rhizobium leguminosarum T88*	Crosslinking process using calcium chloride, calcium gluconate, and calcium lactate	Encapsulation process	Cell survival evaluated before and after drying; storage stability; SEM	---	---	---	---	---	Chaparro-Rodríguez, 2023 [94]

Starch/chitosan	Biofertilizer	Encapsulated *A. brasilense AbV5, AbV6 strains*	Physical crosslinking by chain entanglement	Swelling equilibrium method	FTIR; FEG-SEM; NMR; encapsulation and survivor ratio of A. brasilense	Seeds of maize	Germination test; plant growing evaluation: shoot fresh weight; chlorophyll	Sieved soil and sand	---	---	Lima-Tenório, 2023 [95]
Rice-cooked wastewater (starch), acrylamide, and 2-acrylamido-2-methylpropansulfonic acid	Fertilizer	Urea	Crosslinking using APS as the initiator	Swelling equilibrium method	XRD; FTIR; XPS; Raman spectroscopy; TGA; water absorption; urea solution absorbency; biodegradation; swelling	Chili plants, mung beans (Vigna radiata), and pea seeds	Germination using Chili plants, mung beans (*Vigna radiata*), and pea seeds	Agricultural soil	Mixed with soil	---	Kolya et al., 2023b [45]
Acetylated starch–polyacrylamide	Fertilizer	Urea; potassium nitrate, ammonium sulfate, biofertilizer	Crosslinking using MBA and APS as the initiator Microwave irradiation was also considered	Swelling equilibrium method	FTIR; FE-SEM; XRD; XPS; TGA; swelling test; fertilizer loading; compound release; diffusion; biodegradation: vermi-compost, soil burial and sewerage	.---	.---	.---	.---	.---	Singh, 2023 [82]
Polyvinyl alcohol/humic acid	Fertilizer	Urea	Coating processing by dipping or spraying glutaraldehyde Crosslinking using	Swelling equilibrium method	FTIR; TGA; compression test; SEM; swelling; water retention in soil; compound release; biodegradability	Seeds of sorghum (*Sorghum sp.*)	Germination test; dry weight of root; dry weight of aerial part	Commercial garden soil	.---	.---	Torres-Figueroa et al., 2023 [20]
Composite based on attapulgite; N-isopropylacrylamide; sodium alginate	Fertilizer	Urea	Crosslinking process using MBA and APS as the initiator	Swelling equilibrium method; *in situ* method	FTIR; XRD; TGA; SEM; water absorption; water retention; swelling; compound release	.---	.---	.---	.---	.---	Wu et al., 2023 [90]

Quaternary ammonium guar gum and humic acid	Fertilizer	Humic acid	Not mentioned	Swelling equilibrium method	NMR; FTIR; XPS; TGA; SEM; SECMALS; swelling; apparent viscosity; compound release; UV VIS, biodegradability; water absorption; water retention in soil	Mung beans	Fresh root biomass; fresh leaf/stem biomass; length, root length, and leaf area	.---	.---	.---	Cui et al., 2024 [92]
Pectin hydrogel; pectin-activated carbon hydrogels	Biostimulant	Pectins	Coacervate crosslinking using citric acid	*In situ* method	FTIR; SEM; XRD; Raman; BET; rheological properties; water retention; TGA; mechanical properties; swelling	Mung beans	Seed germination	Soilless cultivation			Kanagalakshmi et al., 2024 [96]
Sodium alginate–humic acid–NIPAm–AMPS N-isopropylacrylamide and 2-acrylamide-2-methylpropanesulfonic acid as monomers, poly(ethylene glycol) dimethacrylate as a crosslinking agent, and sodium alginate and humic acid as filled biomolecules.	Fertilizer	Urea sodium alginate and humic acid	Not mentioned	Swelling equilibrium method	FTIR; SEM; XRD; swelling test; rheology; water retention; compound release; UV VIS; compound release in soil	.---	.---	.---	.---	.---	Hua et al., 2024 [32]
Alginate	Biofertilizer	Microbial consortium: *Arbuscular mycorrhizal fungi* (AMF) and *Azospirillum brasilense Sp7*	Crosslinking by the use of Ca^{2+} ions	Encapsulation process	Spore germination and bacterial growth	*Brachypodium distachyon.*	Plant-host colonization	.---	.---	.---	Mafune et al., 2024 [52]

Natural rubber latex/cassava starch	Fertilizer	Urea	Not mentioned	Encapsulatio n process	FTIR; SEM; bead size; liquid retention; compound release into water; compound release into soil; UV VIS	Thai eggplant	Plant growing: Canopy width; leaf width; leaf length	Loamy soils	.---	.---	Phansroy et al., 2024 [91]
Nanocellulose	Fertilizer	Potassium dihydrogen phosphate; potassium nitrate	Not mentioned	Swelling equilibrium method	Chemical composition; FTIR; TEM; SEM; XRD	Flaxseed	Germination: Number of germinated seeds, stem size	.---	.---	.---	Rodríguez-Quesada et al., 2024 [97]
Gum Arabic-GMA	Biofertilizer	Encapsulation of *A. brasilense* FP2	Not mentioned	Encapsulatio n process	Swelling; encapsulation of *A. brasilense* and survival assays; FTIR; FEG-SEM	Maize (*Zea mays*)	Mean root diameter; high-resolution digital images of maize roots				Lima-Tenório et al., 2024 [100]
Sodium alginate/sodium lignosulfonate	Fertilizer	Humic acid	Crosslinking by the use of Ca^{2+} ions	Swelling equilibrium method	FTIR; XRD; SEM; SLS; swelling; compound release; water-holding capacity; maximum soil water-retention capacity	Maize; pak choi	Seed growing: Plant height	Plantatio n forest soil	.---	.---	Zhang et al., 2024 [34]
