# Supplementary material for: Analysis of the Growth of Hydrogel Applications in Agriculture: A Review
Source: Gels. 2025 Sep 11;11(9):731. doi: 10.3390/gels11090731 (PMC12469933; doi:10.3390/gels11090731)
Supplement: Supplementary file 1 [file gels-11-00731-s001.zip › Table S3.pdf]

Table S3. Documents classified in the Soil conditioners category.

Hydrogel materials	Soil improvement	Hydrogel preparation or crosslinking process	Characterization of materials	Plant parameter		Soil parameter			References
				Plant	Germination or growth	Soil type	Soil/Hydrogel	Soil analysis	
Sodium carboxymethylcellulose	Soil water management	Crosslinking process by slight radiation	Pseudo-gel fraction; swelling	---	---	---	---	---	Liu et al., 2005 [31]
Poly(acrylic acid-co-acrylamide)/$AlZnFe_2O_4$–potassium humate composite	Soil structure; soil water management	Casting process Thermal process: Using thermal cycles	EDS; FTIR; water retention in soil	Wheat (*Triticum aestivum L.*)	Germination percentage; plant length; fresh plant biomass; total dry biomass; shoot dry biomass	Sandy loam soil	Mixed with soil	Soil pH; electrical conductivity; porosity; bulk density; hydraulic conductivity; permanent Wilting point; total available water	Shahid et al., 2012 [47]
Poly(acrylic acid)-graft-mannan copolymer	Microbial activity	Crosslinking process using slight radiation and as the initiator ammonium persulphate	Swelling; viscoelastic micromechanical test; Field emission cryo-SEM; Bacterial strains and growth conditions using *Bacillus subtilis ATCC 6051 T and Pseudomonas fluorescens ATCC 13525*	---	---	Surface soil from low-fertility, coarse-textured wheat field	Mixed with soil	Microbial populations	Pham et., 2017 [113]

Commercial product: Not specified	Soil water management	Commercial product	---	Maize plants (*Zea mays L.*)	Photosynthesis; transpiration; stomata conductance; chlorophyll content; fresh plant biomass; dry plant biomass; fresh forage yield; dry forage yield	Soil used in cultivar to grain and forage production	Mixed with soil	Moisture content	Sandoval et al., 2017 [117]
Super absorbent hydrogels: Commercial product	Microbial activity	Commercial product	Retained soil water available for plant	Perennial ryegrass (*Lolium perenne L.*).	Plant survival rate; shoot fresh biomass; shoot and leaf dry biomass; leaf relative water content; chlorophyll content; leaf relative electric conductivity; leaf oxidative damage	Vermiculite, sand and field top soil	Mixed with soil	---	Su et al., 2017 [1]
Stockosorb 660-medium: Commercial product	Soil water management; microbial activity	Commercial product	---	Olive plantlets (*O. europaea* L. cv. Chemlali	Leaf relative water content; photosynthetic pigment; chlorophyll content; plant dry biomass; total phenols from leaves; mycorrhizal colonization; stomata and trichome densities; leaf relative water content	Sandy P-poor soil	Mixed with soil; R. irregularis were introduced directly below the roots	Initial and final soil moisture	M'barki et al., 2018 [2]
Poly(acrylic acid)-graft-agar/gum Arabic (Agar/Ga-g-PAA)	Soil water management	Grafting of acrylic acid onto gum Arabic/agar using APS initiator and MBA crosslinker under microwave irradiation	Swelling, grafting percentage; FTIR; XRD; SEM)	---	---	Eight different soils collected from the various regions o	Mixed with soil	Water evaporation ratio of soil	Hasija et al., 2018 [107]
Chitosan/potato starch blend	Microbial activity	Crosslinking with STPP	NMR; XRD; TGA; SEM; swelling; cells immobilization; bacterial survival quantification; cells release kinetics in sterile water; cells release kinetics in soil	---	---	Soil from cultivar free of vegetation	Mixed with soil	---	Perez et al., 2018 [10]
Poly(acrylic) hydrogel with protein filler from cattle hide composite	Soil structure	Free-radical copolymerization of neutralized acrylic acid with MBA crosslinker	FTIR; DSC; TGA; water absorption; swelling	---	---	---	---	---	Baidakova et al., 2019 [108]

Stockosorb 660-medium: Commercial product	Soil water management	Commercial product	---	Olive plantlets (*O. europaea* L. cv. Chemlali	Leaf relative water content; photosynthetic pigment; chlorophyll content; plant dry biomass; total phenols from leaves; mycorrhizal colonization; stomata and trichome densities; leaf relative water content; proline content	Sandy P-poor soil	Mixed with soil	---	M'barki et al. 2019 [42]
Poly(acrylamide-co-acrylate) hydrogel with acrylamide process waste	Soil water management	MBA as a crosslinking agent	Water absorption; swelling; fungicidal and antibacterial protection against late blight (*P. infestans*) and black leg (*P. atrosepticum*)	Red Scarlett potato	---	Not specified	Mixed with soil	Soil texture: water permeability; water retention; dispersity; hydraulic conductivity	Smagin et al., 2019 [105]
Polyacrylamide	Bioavailability in soil	TEMED as catalyst and APS initiator agent	---	*Lactuca sativa L. cv. Batavia*	Bioconcentration factor (BCF)	Soil from peri-urban area and soil mixing with compost	---	Soil Hg content	Turull et al., 2019 [106]
Sodium alginate	Microbial activity	Crosslinking using calcium chloride	SEM; XRD; water uptake; EDS	---	---	Sand; calcareous soil; clay	---	Compression test	Taha et al., 2020 [114]
Bacterial alginate-based water-soluble hydrogel	Soil structure	---	Transmission light microscopy	---	---	Coarse quartz sand	Mixed with soil	Uniaxial compression test; hydraulic conductivity	Barrientos-Sanhueza et al., 2021 [44]
Bacterial cellulose interconnected with glycerol–succinic anhydride–EGDE prepolymer	Soil structure	Prepolymer based on glycerol–succinic anhydride–ethylene glycol diglycidyl ether mixed with bacterial cellulose	SEM; TGA; Mechanical parameters; fluorescence parameters	---	---	---	---	---	Ding et al., 2021 [109]
Poly(acrylic acid)-grafted coconut fiber (PAA-g-CF) hydrogel composite	Soil water management	Crosslinking using MBA as crosslinker and APS as the initiator	Swelling; water absorbency; reswelling; FTIR; XRD; EDX	---	---	---	Mixed with soil	Water retention	Saha et al., 2021 [110]

Ca-bacterial alginate hydrogel	Soil structure	Crosslinking by the use of Ca^{2+} ions	FTIR; water soluble	---	---	Coarse quartz sand and kaolinite	Hydrogel dispersion mixed with soil	Uniaxial compression; aggregate stability; hydraulic conductivity; bright field light microscopy	Barrientos-Sanhueza et al., 2022 [104]
Laponite-reinforced poly(acrylic acid) nanocomposite	Soil water management	Ion crosslink	FTIR; swelling; TEM; XRD; cytotoxicity; water retention in soil	Cucumbers (*Cucumis sativus 'Bae Tae Rang'*) and tomatoes (*Solanum lycopersicum 'Mini Chal'*) seeds	Plant height; leaf length; leaf width; leaf number; chlorophyll content; stem diameter, shoot and root fresh biomass; dry plant biomass	River sand	Mixed with soil	Moisture content	Kim et al., 2022 [48]
Amidated pectin	Microbial activity	Crosslinking by the use of Ca^{2+} ions	Cell viability; SEM	---	---	---	---	---	Chaparro-Rodríguez et al., 2023 [94]
Porous Ca-alginate hydrogel from bacterial and algal alginate sources	Soil structure	Not mentioned	Contact angle	---	pH; electrical conductivity; unconfined uniaxial compression; water retention for soil; hydraulic conductivity; bright field light microscopy; mechanical breakdown	Coarse quartz sand	Mixed with soil	---	Dorochesi et al., 2023 [54]
Starch-grafted poly(AM-co-AMPS) hydrogel embedded in waste face masks	Soil water management	Not mentioned	Water absorbency; pH, water retention	Germination using *Pisum sativum* and mung bean (*Vigna radiata*)<br>Plant growth using *Ophiopogon japonicus*	Leaf color	Garden soil	Mixed with soil	---	Kolya et al., 2023a [111]

Rice-cooked wastewater (starch), acrylamide, and 2-acrylamido-2-methylpropansulfonic acid	Soil water management	---	XRD; FTIR; XPS; Raman spectroscopy; TGA; water absorption; urea solution absorbency; biodegradation; swelling	Germination using Chili plants, mung beans (*Vigna radiata*), and pea seeds	---	Cultivated soil	Mixed with soil	---	Kolya et al., 2023b [45]
Aldehyde-chitosan/Cu-doped carbon dot/KCl (COCu-K)	Mulch	Crosslinking by the use of Cu^{2+} ions	FTIR; FE-SEM; XRD; XPS; TGA; swelling; water absorption and retention ability; soil degradation	Weed seeds and Chinese flowering cabbage (Brassica campestris L. ssp. chinensis var. utilis Tsen et Lee) Seeds of pepper (Capsicum annuum L. var. conoides)	Weed germination; height of each plant	Pasture soil extracted from farmland	Mixed with soil	---	Li et al., 2023 [115]
Modified potassium carbonate	Soil structure	Not mentioned	---	---	---	Cultivated soil	Mixed with soil	Soil aggregate stability	Mayerová et al., 2023 [116]
Xanthan gum/cellulose fibers composite	Soil water management	Crosslinking by thermal process	Rheology; FTIR; FE SEM; light microscopy; water absorption; soil degradation	Grass seeds of *Nepeta Cataria* and tomato plants (*Solanum lycopersicum var. Cerasiforme*)	Seed germination Vegetation mass of plants	Forest soil	Mixing with soil	Water-holding capacity, water retention of soil; SEM	Sorze et al., 2023 [112]
Hydrogel based on CMC–Na, SA and CS	Soil structure	Not mentioned	Swelling; FTIR; XPS; SEM; TGA-DSC; compound release	---	---	Moderately saline-alkali soil, highly saline soil, and highly alkaline soil	Mixing with soil	pH; electrical conductivity; soil cation exchange capacity; exchangeable sodium content; water-soluble salt content; organic matter; total nitrogen; total phosphorus; available phosphorus	Qi et al., 2024 [33]
M-BFA/DMAA	Soil water management	Crosslinking using MBA and ammonium dithionite as the initiator	BET; SEM; XPS; swelling; water retention	---	---	Sand and non-specified soil	Mixing with soil	---	Shi et al. 2024 [36]

Agarose-bacterial cellulose composite	Soil water management	Crosslinking using MBA	FTIR; XRD; SEM; TGA; swelling	Alfalfa seeds	Seed germination	---	---	---	Tang et al., 2024 [55]
