# Supplementary material for: Analysis of the Growth of Hydrogel Applications in Agriculture: A Review
Source: Gels. 2025 Sep 11;11(9):731. doi: 10.3390/gels11090731 (PMC12469933; doi:10.3390/gels11090731)
Supplement: Supplementary file 1 [file gels-11-00731-s001.zip › Table S4.pdf]

## Table S4. Documents classified in the Bioactive category

| Hydrogel materials | Activity | Hydrogel preparation or crosslinking process | Compound loading | Characterization of materials | Plant parameter | | Soil parameter | | | References |
|---|---|---|---|---|---|---|---|---|---|---|
| | | | | | Plant | Germination or growth | Soil type | Soil/Hydrogel | Soil analysis | |
| Methyl-esterified pectin/humic substances | Phloroglucinol as model molecule for phenols | Crosslining using calcium chloride | Swelling equilibrium method | Solid-state NMR; SEM; MRI; rheology; phloroglucinol release as model molecule for phenols | --- | --- | --- | --- | --- | Nuzzo et al., 2020 [121] |
| Se-F, Se-H, and Se-T collagen–starch | Bioactive plant support system | Crosslinking using polyurethane | Seed encapsulation | SEM; EDS; FTIR; WAXS; UV-Vis | Tomato (Solanum lycopersicum) | Proliferation of tomato cells by epifluorescence microscopy | Commercial soil for vegetables | Mixed with soil | --- | Flores-Urquieta et al., 2024 [58] |
| Mo-bioMOFs/amino acids/collagen and starch | Bioactive plant support system | Crosslinking using polyurethane | Seed encapsulation | SEM; EDS; FTIR; WAXS; UV-Vis | Tomato (Solanum lycopersicum) | Proliferation of tomato cells by epifluorescence microscopy | Commercial soil for vegetables | Mixed with soil | --- | Valdés-Lozano et al., 2024 [122] |
